# Transcriptome Analysis Reveals New Insight of Fowl Adenovirus Serotype 4 Infection

**DOI:** 10.3389/fmicb.2020.00146

**Published:** 2020-02-11

**Authors:** Yuan Chen, Ruiling Huang, Guishu Qu, Yaoshun Peng, Lihui Xu, Changkang Wang, Cuiqin Huang, Quanxi Wang

**Affiliations:** ^1^College of Animal Science (College of Fee Science), Fujian Agriculture and Forestry University, Fuzhou, China; ^2^Fujian Key Laboratory of Traditional Chinese Veterinary Medicine and Animal Health, Fujian Agriculture and Forestry University, Fuzhou, China; ^3^Fujian Provincial Key Laboratory for the Prevention and Control of Animal Infectious Diseases and Biotechnology, Longyan, China

**Keywords:** transcriptome, fowl adenovirus serotype 4, virus genome, viral invasion, intracellular trafficking

## Abstract

Since 2015, Fowl adenovirus serotype 4 (FAdV-4) infection has caused serious economic losses to the poultry industry worldwide. We isolated and identified the FAdV-4 strain NP, from infected chickens on a layer farm, using chicken embryo allantoic cavity inoculation, electron microscopy, viral genome sequencing, and regression analysis. To explore the pathogenesis of FAdV-4 infection, we conducted transcriptome sequencing analysis of the liver in chickens infected with FAdV-4, using the Illumina® HiSeq 2000 system. Two days after infection with the FAdV-4 NP strain, 13,576 differentially expressed genes (DEGs) were screened in the liver, among which, 7,480 were up-regulated and 6,096 were down-regulated. Gene ontology (GO) analysis indicated that these genes were involved in 52 biological functions. Furthermore, Kyoto Encyclopedia of Genes and Genomes (KEGG) analysis showed that those DEGs were involved in 33 pathways. We then focused on the KEGG pathway of phagosome and found that mRNA levels of the 25 DEGs in that pathway were up-regulated, and seven DEGs were down-regulated. Real-time quantitative polymerase chain reaction (qPCR) confirmed the accuracy and reliability of these findings. Moreover, 24 h after LMH cells were infected with FAdV-4, the mRNA levels of *F-actin, Rab7, TUBA*, and *DVnein* were significantly increased. These four genes were all subsequently silenced by RNA interference, and viral replication of FAdV-4 was then significantly down-regulated. These findings demonstrate the isolation and identification of the FAdV-4 NP strain, and the DEGs in KEGG pathway of phagosome were utilized by FAdV-4 to benefit its infection.

## Introduction

Based on the genomic profiles, Fowl adenovirus (FAdV) is divided into three groups (I–III) and 12 serotypes (Hess, 2000). It is a non-encapsulated, double-stranded DNA virus (Griffin and Nagy, 2011). Its main structural proteins include the penton, hexon, and fiber proteins (Shah et al., 2017). Infection with different serotypes of group I FAdV leads to different clinical symptoms. Infection with FAdV serotype 4 (FAdV-4) causes hydropericardium hepatitis syndrome (HHS) (Asthana et al., 2013). Broilers at 21–35 days old are easily infected with FAdV-4. The morbidity and mortality rates are high (Chandra et al., 2000). In 1987, FAdV-4 was first reported in Pakistan, and then in India (Mittal et al., 2014) and South Korea (Kim et al., 2008), among other countries. Before 2014, only sporadic cases had been reported in China. Since 2015, FAdV-4 infection has caused a major outbreak in China. However, little is known about the pathogenic mechanisms of FAdV-4 infection.

Viruses evade the defense responses of host in order to efficiently infect host cells. Furthermore, viruses often utilize the host cell machinery to replicate, spread, and survive (Toscano and de Haan, 2018). Receptors are located on the cytomembranes or endosomal membranes. The receptors are internalized within endosomes, from which they are transferred to lysosomes for degradation, while others are recycled and transported back to the plasma membrane. After binding with the receptor, viral structural proteins serve as pathogen-associated molecular patterns that bind to toll-like receptors (TLRs). Some viral particles could inhibit the activation of TLRs to evade the proteasome, and the antigen presentation to cells of the host's immune system during the initial stages of infection (Horvath et al., 1992).

In this study, we identified FAdV-4 strain NP from sick layers that were pre-laying Hy-Line Variety Brown hens and investigated the transcriptomic profile of livers infected with FAdV-4 strain NP. We then focused on the KEGG pathway of phagosome and used qPCR and ribonucleic acid (RNA) interference to determine whether the KEGG pathway of phagosome was used to facilitate FAdV-4 invasion.

## Materials and Methods

### Compliance With Ethical Standards

The animal protocol used in this study was approved by the Research Ethics Committee of the College of Animal Science, Fujian Agriculture and Forestry University. All experimental procedures were performed in accordance with the Regulations for the Administration of Affairs Concerning Experimental Animals, approved by the State Council of China.

### Main Reagents

The PEASY-T1 cloning vector kit, total RNA extraction kit, and reverse transcription kit, qPCR kit, Trans1-T1 competent cells (Full-type Golden Biotechnology Co., Ltd., Beijing, China), and agar rapid recovery kit were purchased from Tiangen Biochemical Technology Co., Ltd. (Beijing, China). The deoxyribonucleic acid (DNA) extraction kit was purchased from Promega Biotechnology Co., Ltd. (Beijing, China). The hematoxylin and eosin (H&E) stains were purchased from Nanjing Jiancheng Technology Co., Ltd. (Nanjing, China), whereas the Dulbecco's modified Eagle medium (DMEM), fetal bovine serum, and the real-time fluorescent quantitative polymerase chain reaction kit were purchased from TaiJing Biotechnology Co., Ltd. (Xiamen, China).

### Embryonated Eggs

Specific-pathogen-free (SPF) embryonated eggs were provided by Jinan Sais Poultry Co. Ltd. The eggs were hatched to 10 day-old in an incubator at 37°C.

### Virus Isolation

The virus was isolated from diseased layers in Nanping city of Fujian province. The liver from each of the infected layers was collected, ground with sterile phosphate-buffered saline (PBS, pH 7.2), and centrifuged at 10,000 g for 10 min. After the supernatants were filtered through a 0.22-μm syringe-driven filter, they were inoculated into the allantoic cavities of 10-day-old SPF embryonated eggs, and cultured in an incubator at 37°C. After inoculation, the embryonated eggs were checked daily and the allantoic fluid was collected from days 1–3. The virus was propagated through more than three passages. The harvested allantoic fluid was frozen at −80°C for viral DNA extraction and subsequent observation using electron microscopy. The median embryo lethal dose (ELD_50_) was calculated using the Reed-Muench method (Wang et al., 2014). The virus was named FAdV4 strain NP.

### Observation by Electron Microscope

The allantoic fluid from the infected embryonated eggs was again centrifuged at 10,000 g for 45 min. The virus was suspended in ultrapure water, and then doubly treated with 2.5% glutaraldehyde for 2 h at 4°C, and 1% osmic acid fixation for 1 h. It was then embedded in epoxy resin and sliced into 50–60 nm sections, and then stained with 3% uranium acetate-citric acid. The morphology of the virus was observed using an HT7700 electron microscope (Wang et al., 2019).

### Identification by PCR and Sequencing of PCR Products

Specific primers based on the A fragment of the hexon gene (AJ554049.1) were designed as follows: F:5′-TGGACATGGGGGCGACCTA-3′ and R:5′-AAGGGATTGACGTTGTCCA-3′. They were then sent to Shanghai Sangon Biological Co., Ltd. for synthesis.

The DNA of FAdV4 was extracted using a viral DNA extraction kit (Promega Biotechnology Co. Ltd., Beijing, China). The PCR was then conducted with specific primers for the A fragment of the hexon gene. The PCR reaction system (25 μL) comprised 1 μL of template, 12.5 μL of PCR Nucleotide Mix (10 mmol/L), 9.5 μL of nuclease-free water, and 1 μL of each of the upstream and downstream primers. The reaction conditions were as follows: denaturation at 95°C for 30 s, annealing at 55°C for 30 s, and extension at 72°C for 30 s, over 35 cycles. After amplification, the PCR products were analyzed via 1% agarose gel electrophoresis. Then the PCR products were sent to sequence by Sangon Biotech (Shanghai) Co., Ltd. At last, the genetic tree was analyzed by DNAMAN9.0 software (Lynnon Biosoft, San Ramon, USA).

### Sequencing Analysis of the FAdV4 Genome

The FAdV-4 strain NP [median tissue culture infective dose (TCID_50_) = 10^−6.23^/0.01 mL] was multiplied in a chick embryonic fibroblast cell line (DF-1) with 5% fetal bovine serum in Dulbecco's modified Eagle medium (DMEM; Hyclone, Logan, USA). At 24 h post-infection, the cells were collected, and viral DNA was extracted with a viral DNA extraction kit (Promega Biotechnology Co., Ltd., Beijing, China). The DNA sample was sent to Wuhan Thermofly Co. Ltd. for sequencing (PE150, HiSeq X™ Ten, Illumina, USA). The genomic library of FAdV4 was constructed with 200–300 bp fragments, which were amplified by PCR with specific primers (shown in Table 1) based on the reference genome of FAdV-4 strain CH/AHMC (GenBank: KU558760.1). The overlap between the amplified bands was about 50 bp. All PCR products were further evaluated for end repair, A-tailing and adapter ligation. The genomic library of FAdV4 was sequenced (PE150; Hiseq X ten, Illumina, USA).

**Table 1:** Special primers of the FAdV 4 genome.

**Genes**	**Sequences (5^**′**^-3^**′**^)**
V1	F:5′-CATCATCTTATATAACCGCGTCTTT-3′ R:5′-CGGTCCGAAGCCATCTGTAAT-3′
V2	F:5′-GAACTGGCTGCGATGCTAGA-3′ R:5′-AAGAGCGACTACACGCGAAA-3′
V3	F:5′-TGTTTCGTCCTTGCACGCAT-3′ R:5′-ACAACGCCAGAGTAGCATCC-3′
V4	F:5′-AGCATTTTGGCGTTGGAGTG-3′ R:5′ACTGTGTTCGGGTTCTAGCG-3′
V5	F:5′-GAGAGTGGGGACGTTACCGA-3′ R:5′-TGTCACGCTTCTCAACGACA-3′
V6	F:5′-GTCATCGCTCTCTTCTGGGG-3′ R:5′-ACAGGTAGCGAAATGTGGCA-3′
V7	F:5′-GGTCTCCGCTGAGCATGAAA-3′ R:5′-CTTTCAACGCCGACGATCAC-3′
V8	F:5′-CAGCGAACACTTCCGATCCA-3′ R:5′-AACTCATCCTCCTCTCCGCT-3′
V9	F:5′-AGAAGCGGATGAGCGAGATT-3′ R:5′-CAACTGGATGGACTCGGTGG-3′
V10	F:5′-CCATAGCAATTTCCGCACCA-3′ R:5′-CAGGGACGGTACAAAGTCGT-3′
V11	F:5′-CTACCGTCGCCTCCATCTAC-3′ R:5′-GGTGTTGGCGTTTTGTACCC-3′
V12	F:5′-CGAGCACCTCCAAAGACACG-3′ R:5′-TCCACAGGTAGTTGTCGCAG-3′
V13	F:5′-AACTCTGGGTTTATCGCCCC-3′ R:5′-CGGTATGCGCGAAAGACAAG-3′
V14	F:5′-TCCAACTCAGGTCGTGCTTC-3′ R:5′-GTCCCCCATCTGCCGAATAG-3′
V15	F:5′-TTGTCTAGGTAGGCGTTGGC-3′ R:5′-TTGTCTAGGTAGGCGTTGGC-3′
V16	F:5′-GAACTGCTCGCCATCAACAC-3′ R:5′-GCTGGGACCTTGAACACGTA-3′
V17	F:5′-GCAATTATCGGGCGGACTGA-3′ R:5′-GGGGGAGTAGACGTAACCCT-3′
V18	F:5′-TAGCCTTCACTGTCTCGCTG-3′ R:5′-CTCCCCTGTCACTACTTGCG-3′
V19	F:5′-CTTTGTTCCCGCGTCCAATC-3′ R:5′-ATTAGGTCGCGCATTCCGAT-3′
V20	F:5′-CCGAAACCGAACTCACCGAT-3′ R:5′-TGTATAACGGAGGAGGCGGA-3′
V21	F:5′-CATGGACCTTGTCCTGTTTGTT-3′ R:5′-TAACGAGGGATACGCGCAAG-3′
V22	F:5′-CGCTCCGAACACAAGGTACA-3′ R:5′-CGAAGGGTGAGAGCTGGTTT-3′
V23	F:5′-TATGCTGAGAACCCTCCCCAA-3′ R:5′-GATCGGTTCCGTGCTGTTTT-3′
V24	F:5′-ACCTCAGAACCAACTACTGCTG-3′ R:5′-CATCATCTTATATATAACCGCGT-3′

### Animal Infection

Eighteen 40-day-old chicks (FAdV4 antigen and antibody negative) were randomly divided into the control group or infection group. Chicks in the infection group were administered an intramuscular injection of 0.5 mL FAdV4 (TCID_50_ = 10^−6.23^/0.1 mL), and those in the control group were injected with the same volume of sterile saline.

### Observation of Pathological Changes

When clinical signs were apparent in the infected group, the birds were sacrificed, and sections of the heart, liver, and kidney of each were collected, stained with H&E and subjected to pathological evaluation.

### Transcriptome Sequencing and Screening of Differentially Expressed Genes (DEGs) in the Liver

At 2 days post-infection with FAdV4, the livers in the two groups were collected and total RNA was extracted using an RNA extraction kit (Saffer, China). The mRNA was separated with magnetic beads to generate a sequence library using the Illumina® HiSeq TM 2000 (Illumina, Shenzhen, China). The concentration of total RNA was quantified with a NanoDrop® 1000 spectrophotometer (OD260/280 = 1.8–2.0) (Thermo Scientific, Shanghai, China) and its purity assessed with electrophoresis in 1% (w/v) agarose gel, which was to detect whether the RNA was degradation or was contaminated with proteins and eoxyribonucleic acid (DNA) (Wang et al., 2015).

We then evaluated the quality of sequenced reads and analyzed their saturation following as our previous study (Wang et al., 2015). Sequencing results of Illumina ®HiSeq TM 2000 is raw reads, in which the impurities as reads with adapter, need to be removed. After removing the impurities, the reads is clean reads. The clean reads should be further filtrated to high quality clean reads, in which the reads with adapter, reads containing N more than 10% were further removed. The map of classification of clean read was present.

Then first, the high quality clean reads were compared with sequences of ribosome using the short-read comparison software Bowtie. Second the ribosome reads were removed. The clean reads-removed ribosome reads were compared with avian genomes and gene sequences using the software TopHat2. Third, the sequencing saturation was used to assess whether the sequencing amount of the sample was saturated. Because when the sequencing amount reaches a certain value, the rate of transcription tends to be gentle, indicating that it is saturated. The data was present with saturation curve.

The number of genes expressed was calculated as the reads per kilobase per million mapped reads (RPKM) and genes were compared to determine their relevant features. The differential test was then conducted and the multiple hypothesis tests were calculated by the detection method based on sequencing of DEGs (Audic and Claverie, 1997). The field value of P was controlled by the false discovery rate (FDR). The DEGs were defined as those for which the FDR was ≤ 0.05 and its difference ratio was <2-fold (Wang et al., 2015; Wang Q. X. et al., 2018).

Gene Ontology (GO) analysis and Kyoto Encyclopedia of Genes and Genomes (KEGG) pathway analysis of DEGs. The DEGs were first mapped to the respective terms of the GO database (http://www.geneontology.org/), and the number of genes per term was calculated. A list of genes was thereby obtained with specific numbers and GO functions. The hypergeometric test was then applied to find GO entries that were significantly enriched in the DEGs compared with the entire genomic background.

The corrected *p* ≤ 0.05 was used as the threshold, and the GO term satisfying this condition was defined as the significantly enriched GO term in the differentially expressed transcript (Wang et al., 2015; Wang Q. X. et al., 2018).

### KEGG Enrichment Analysis

The KEGG enrichment analysis was applied to find the pathways that were significantly enriched in the differentially expressed transcripts compared with the whole genome. Pathways with a *Q* ≤ 0.05 were defined as significantly enriched pathways in the differentially expressed transcripts. Significant pathway enrichment was able to identify the most important biochemical metabolic pathways and signal transduction pathways involved in the differentially expressed transcripts.

### Fluorescence Quantitative PCR

In this study, we focused on the pathways associated with the process of viral infection. The mRNA expression of genes in these pathways were determined by qPCR, using specific primers (shown in Table 2), which were designed based on the sequences in Genebank (β-actin: X00182.1, CR: XM_015289135.2, αMβ2: XM_015289136.2, αVβ3: XM_015299304.2, MR: XM_015853441.1, F-actin: NM_001308613.2, Rab7: XM_003212919.3, TUBA: NM_001305272.1, DVnein:XM_015281907.2, VAMP-3: NM_001039489.1, VATPase: XM_004935760.3, MHCII:XM_015294966.2, MHCI:HQ141386.1, SRB1:XM_415106.4). At 24 h post-infection with FAdV4, the mRNA expression of those genes was further examined by qPCR. Furthermore, β-actin was used as a reference gene and mRNA levels were calculated using the ΔΔCT method. The mRNA expression of the *hexon* gene of FAdV4 was calculated based on a standard curve.

**Table 2:** Sequences of qPCR primers in this study.

**Genes**	**Sequences (5^**′**^-3^**′**^)**	**Expected size (bp)**
β-actin	F:5′-CCAAAGCCAACAGAGAGAAGAT-3′ R:5′-CATCACCAGAGTCCATCACAAT-3′	138
CR3	F:5′-GCTTTGGCTTTGGTGCATGG-3′ R:5′-CTCCTTCTGTCTTGCGCTGG-3′	108
*αMβ2*	F:5′-GAGACCACCAGTAGCCAGGT-3′ R:5′-AGCACCAGGAGCTAGGACAG-3′	181
*αVβ3*	F:5′-CTCACCAGCAACCTTCGCAT-3′ R:5′-TTGAAGCGCATCACCTCGTC-3′	179
*MR*	F:5′-AGTGTCCCTTGGCTGGACTT-3′ R:5′-CTACAGCGCACGTACTGCAA-3′	161
*F-actin*	F:5′-GCTTCCATCCAGTGCGGAGTATTC-3′ R:5′-GCTTAGAACGGCGGCGACAG-3′	88
*Rab7*	F:5′-CCTGCTGCTTGGATCTGCTCTC-3′ R:5′-CTGTAAGGTGGCTGGCACTGC-3′	108
*TUBA*	F:5′-GCGGCACGGCAAGTACATGG-3′ R:5′-CTTGGTCTTGATGGTGGCGATGG-3′	94
*DVnein*	F:5′-AGATGTTCAAGCAGGAGCCAACC-3′ R:5′-TCCATACAGGTGACAACACGATGC-3′	87
VAMP-3	F:5′-CGGCAGGATGTCAGCCAATGTC-3′ R:5′-TTCCAGCACCTTGTCCACATTCAC-3′	133
*VATPase*	F:5′-GAGGAACTGCTGCGCTCTGTG-3′ R:5′-GCCAGAACTCCGACATGATCCG-3′	85
*MHCII*	F:5′-CATGCTAGCTTGGAGGAGCC-3′ R:5′-GGTAGTTGTGACCGGGAAGC-3′	187
*MHCI*	F:5′-TGGGTGTGGGATGGGCTC-3′ R:5′-GAAACACAGCCGGGCAGG-3′	200
*SRB1*	F:5′-AGATCACCACCAGTCTCCAGAAGG-3′ R:5′-CAGAACGACAGATGAGCCAGCAG-3′	197

### *F-actin, Rab7, TUBA*, and *DVnein* Gene Silencing

The LMH cells were prepared in 6-well plates with DMEM containing 10% fetal bovine serum at 37°C, under 5% carbon dioxide (CO_2_). The F-actin-, Rab7-, TUBA-, and DVnein-specific siRNA oligonucleotides were designed (based on: F-actin: NM_001308613.2, Rab7: XM_003212919.3, TUBA: NM_001305272.1, DVnein:XM_015281907.2) and synthesized by Biomics Biotechnology Co. Ltd. (Nantong, China). The siRNA sequences were as follows: si-F-actin F:CGAUGAAGAUCAAGAUCAUdTdT, R:AUGAUCUUGAUCUUCAUCGdTdT; si-Rab7 F:CCAUGGUGUCGACCUUCUAdTdT, R:UAGAAGGUCGACACCAUGGdTdT; si-DVnein F:GAAAGAUGGAGAUGUUCAAdTdT, R:UUGAACAUCUCCAUCUUUCdTdT; and si-TUBA F:AGCUGGAGUUCUCCAUCUdTdT, R:UAGAUGGAGAACUCCAGCUdTdT. When the siRNA sequences were transfected into LMH cells for 6 h, the cells were subsequently infected with FAdV4. The mRNA levels of *F-actin, Rab7, TUBA, DVnein*, and the hexon gene of FAdV4 were detected by qPCR at 24 h post-infection.

### Statistical Analysis

Statistical analyses were performed via *t*-tests or one-way analysis of variance (ANOVA) and the least significant difference (LSD) test using the SPSS 20.0 software.

## Results

### Isolation and Identification of FAdV4 Strain NP

**Figure 1:**
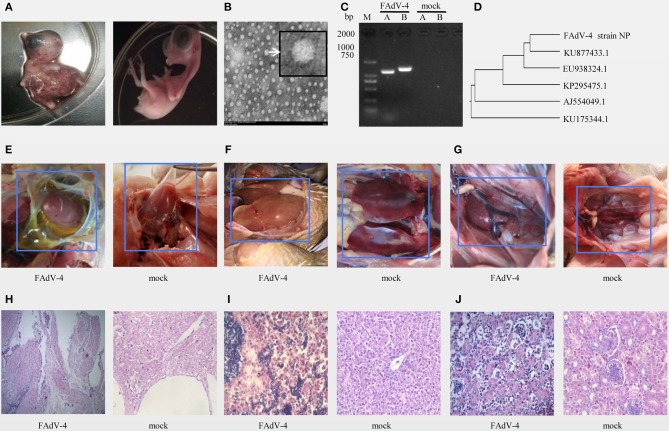
Isolation and identification of FAdV-4 strain NP. Chickens on a layer farm in Fujian province of China were affected by an outbreak of the infectious disease. Ten-day-old specific-pathogen-free (SPF) embryonated eggs were inoculated with 200 μL of filter liquid. (**A**, left) At 24–48 h post-inoculation, the embryos died and showed severe hemorrhage. (**A**, right) The embryonated eggs in control group were inoculated with 200 μL of Sterilized saline. The allantoic fluid of chicken embryos was collected and concentrated. **(B)** The allantoic fluid was examined using electron microscopy (10,000×). **(C)** Viral serotypes were identified by PCR. **(D)** The hexon gene sequence was analyzed using a genetic evolution tree. **(E–G)** Chickens in FAdV-4 infection group (at left) and in control group (at right) were dissected and observed. Tissue sections of the heart (**H**, FAdV-4 infection at left, control group at right), liver (**I**, FAdV-4 infection at left, control group at right), and kidneys (**J**, FAdV-4 infection at left, control group at right) were prepared and stained with hematoxylin and eosin **(H,E)**. Sections were then observed microscopically (400×).

The results of inoculation of 10-day-old SPF chicken embryos showed that they all died 24–48 h after infection, were severely swollen, with oedema and hemorrhage (Figure 1A left), compared with the control embryo (Figure 1A right). Observation of the viral structure by electron microscopy revealed that the virus was 70–90 nm in size, spherical, non-encapsulated, and with a typical icosahedral structure (Figure 1B).

The PCR amplification with specific primers of the A and B fragments of the hexon gene showed specific bands around 1,219 and 1,350 bp (Figure 1C). The sequence of the A fragment of the hexon gene indicated that the virus was FAdV4 (Figure 1D).

In another animal experiment, 8 out of 9 40-day-old SPF chickens died within 3 days of being inoculated intramuscularly with the NP virus (TCID_50_ = 10^−6.23^/0.01 mL), and all chickens of control treatment were alive. Pathological evaluation of the pericardial sac showed that it was filled with transparent or straw-colored fluid (Figure 1E left), the pericardium of the control group had no inflammatory exudate (Figure 1E right). And the liver-infected with FAdV-4 is swollen, embrittlement, and xanthochromia (Figure 1F left), compared with the control group (Figure 1F right). The kidney-infected with FAdV-4 showed swollen with white necrotic foci (Figure 1G left), compared with the control group (Figure 1G right). The pathological sections of hart-infected with FAdV-4 showed that there were many lymphocyte infiltration between the myocardiums (Figure 1H left), compared with hart in the control group (Figure 1H right). In the pathological section of liver-infected with FAdV-4, hepatocytes were present to degeneration and necrosis (Figure 1I left), compared with the livers in control group (Figure 1I right). And the cells in renal tubular-infected with FAdV-4 also showed degeneration and necrosis (Figure 1J left), compared with the kidney in control group (Figure 1I right).

### Genomic Profile of FAdV4 Strain NP

**Figure 2:**
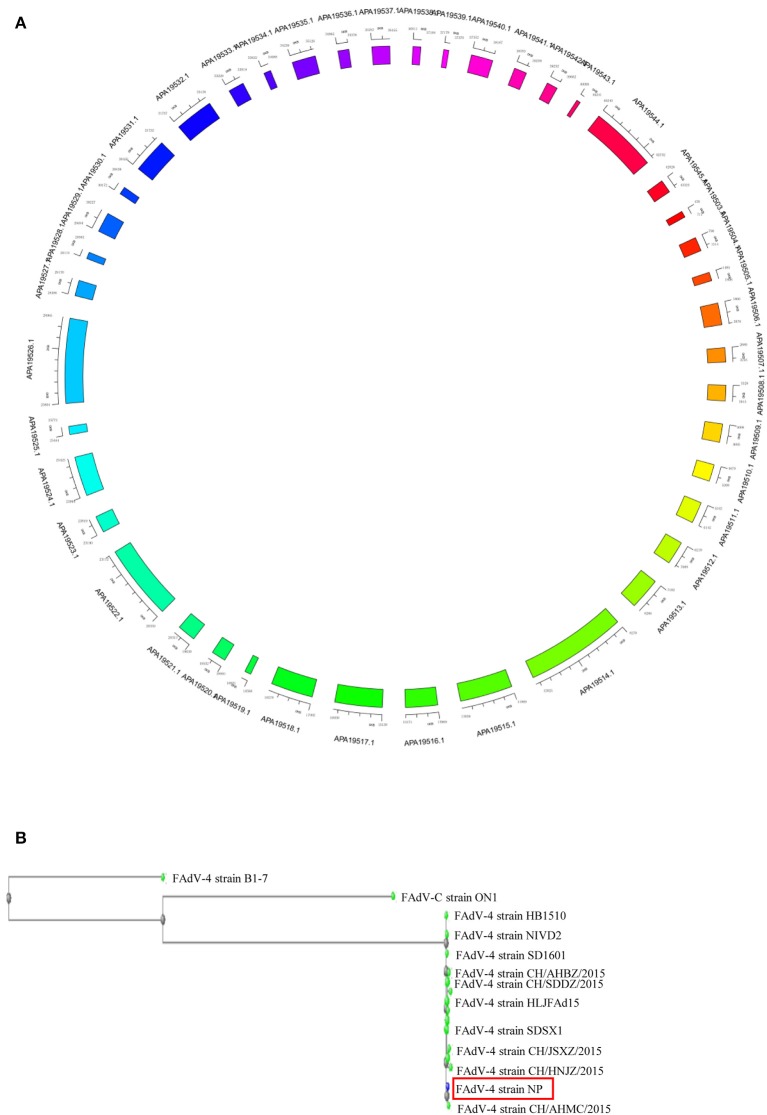
Genome map of FAdV-4 strain NP. The sequences were compiled and edited using the SeqMan program (laser gene) to produce the whole-genome sequence of strain NP. **(A)** The genome was mapped. **(B)** The homology of FAdV-4 strain NP was analyzed through genetic evolution tree.

The complete genome of FAdV4 strain NP was 43,738 bp in length (the sequence has been submitted to Genbank, the submission ID is 2266218) (Supplemental Data 1), with 54.9% G+C content and 42 open reading frames (ORFs) (Figure 2A) (the sequence has been submitted to Genbank, accession numbers MN604703 to MN604744). The FAdV4 strain NP showed a typical genome organization of FAdV-4, with two fiber genes (protein fiber-1 and fiber-2). Compared with the genomic sequences of other published FAdV-4 strains, 98.3–100% homology was observed at the nucleotide level. The NP strain was most closely matched to the CH/HNJZ2015 strain, isolated in China in 2015 (Figure 2B).

### DEG Screening in the Livers Infected With FAdV-4

**Figure 3:**
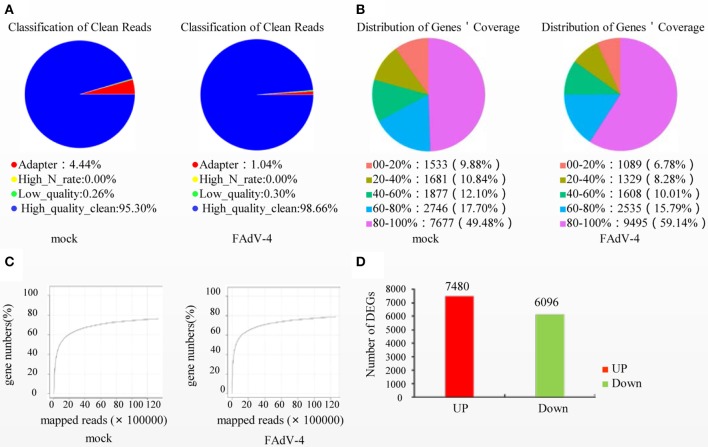
Screening of differentially expressed genes (DEGs) in livers infected with FAdV-4. Two days after infection with FAdV-4, the genes expressed in the livers were subjected to transcription and sequencing analysis. **(A)** Classification of clean reads. **(B)** Distribution of gene coverage. **(C)** Mapped reads were evaluated in FAdV-4 infected livers and control livers. **(D)** Histogram showing screened DEGs.

Based on the results of the sequencing quality evaluation, the high-quality clean reads in the two groups were all higher than 95.00% (Figure 3A). The distribution of gene coverage in the control group was up to 49.48%, and up to 59.14% in the FAdV4-infected group (Figure 3B). The mapped reads were >60% (Figure 3C). These results indicated that the sequencing quality in the livers of the two groups were favorable for further study. In the current study, the genome of Gallus gallus-5.0 (chicken) (GRCg6a, GCF_000002315. 6, https://www.ncbi.nlm.nih.gov/genome/?term=Gallus%20 domesticus) was as our reference genome. A total of 78,924,070 paired reads were detected in the control livers and 78,033,860 paired reads in the livers infected with FAdV-4. The mapping ratio in the control was 85.78%, and in livers infected with FAdV-4, 75.29% (Table 3). And a total of 26,512 transcripts were annotated in the control livers and 28,708 in the livers infected with FAdV 4 strain NP. In the current study, 10,024 novel annotation transcript were found in the control livers and 10,567 in the livers infected with FAdV 4 strain NP (the sequence of novel annotation transcript has been submitted to Genbank, the submission ID is 6338149).

**Table 3:** Genomic alignment results.

**Samples**	**Control group**	**FAdV-4-infected**
Total paired reads, number (%)	78,924,070 (100.00)	78,033,860 (100.00)
Unmapped paired reads, number (%)	11,226,317 (14.22)	19,282,033 (24.71)
Unique mapped paired reads, number (%)	67,322,232 (85.30)	58,369,327 (74.80)
Multiple mapped paired reads, number (%)	375,521 (0.48)	382,500 (0.49)
Mapping ratio (%)	85.78	75.29

A total of 13,576 DEGs were then screened from the livers infected with FAdV-4 and compared with the control livers. These DEGs included 7,480 up-regulated unigenes and 6,096 down-regulated unigenes (Figure 3D).

### GO Functional Classification

**Figure 4:**
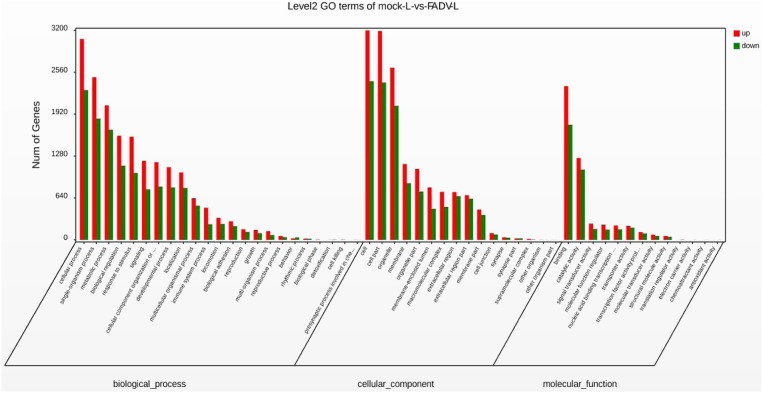
Gene Ontology (GO) functional annotation and classification of differentially expressed genes (DEGs). Functional classification of DEGs was performed using GO analysis (http://www.geneontology.org/) and the categories “biological process,” “molecular function,” and “cellular component” were analyzed.

The GO functional classification of the 13,576 unigenes revealed three categories: “biological processes,” “cellular compounds,” and “molecular function.” As shown in Figure 4, the numbers of up-regulated unigenes and down-regulated unigenes in “cell processes” were highest in “biological processes.” Furthermore, the numbers of up-regulated unigenes and down-regulated unigenes in “cell” were highest in the category of “cellular compounds.” In the category of “molecular function,” the DEGs associated with binding were the highest in number (Figure 4).

### KEGG Enrichment Analysis and Real-Time qPCR Confirmation

**Figure 5:**
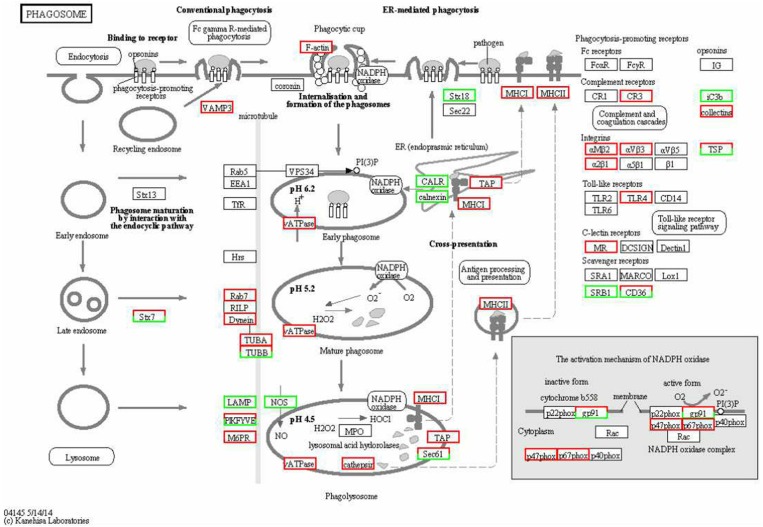
KEGG functional annotation of differentially expressed genes (DEGs) involved in the cell phagosome pathway. A red rectangle denotes that the DEGs are significantly up-regulated [log fold change > 1, false discovery rate (FDR) < 0.001]; A green rectangle denotes that the DEGs are significantly down-regulated (log fold change > 1, FDR < 0.001) in the FAdV-4-infected liver, compared with the control liver.

The KEGG enrichment analysis helped us to further understand the biological functions of DEGs. We found that the *Q*-value of the phagosome was significantly up-regulated (Figure 5). The mRNA levels of *CR3*, α*M*β*2*, α*V*β*3*, α*2*β*1, TLR4, MR, F-actin, Rab7, TUBA, DVnein, VATPase, VAMP-3, MHCI*, and *MHCII* were significantly up-regulated in FAdV-4 infected livers, compared with the control livers.

**Figure 6:**
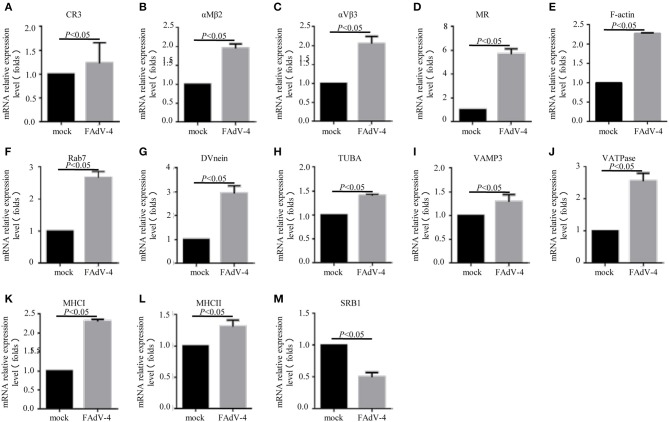
The mRNA levels of genes involved in the phagosome pathway in the liver, as revealed by qPCR. Two days after infection with FAdV-4, the livers were collected and the mRNA levels of *CR3* **(A)**, α*M*β*2* **(B)**, α*V*β*3* **(C)**, *MR* **(D)**, *F-actin* **(E)**, *Rab7* **(F)**, *DVnein* **(G)**, *TUBA* **(H)**, *VATPase* **(I)**, *VATPasw* **(J)**, *MHCI* **(K)**, *MHCII* **(L)**, and *SRB1* **(M)** were detected by qPCR. All data are presented as the mean ± SE of three independent experiments. Significant differences between the treatments were determined by the Student's *t*-test, using the SPSS software (version 20.0). Label of *p* < 0.05 denotes significance between the treatments.

In order to confirm the DEGs levels, we evaluated the mRNA levels of *CR3*, α*M*β*2*, α*V*β*3, MR, F-actin, Rab7, TUBA, DVnein, VAMP-3, VATPase, MHCI, MHCII*, and *SRB1* in livers infected with FAdV-4 strain NP. After infection, the mRNA levels of *CR3*, α*M*β*2*, α*V*β*3, MR, F-actin, Rab7, TUBA, DVnein, VAMP-3, VATPase, MHCI*, and *MHCII* were up-regulated and the mRNA level of *SRB1* was down-regulated (Figures 6A–M). These results were consistent with those of the KEGG analysis (Table 4).

**Table 4:** Results of qPCR verification.

**Genes**	**Results of expression spectrum Up (+)/Down (–)**	**Results of qPCRUp (+)/Down (–)**	**Folds**
CR3	+	+	1.33 ± 0.001
*αMβ2*	+	+	1.95 ± 0.001
*αVβ3*	+	+	2.04 ± 0.001
*MR*	+	+	5.67 ± 0.001
*F-actin*	+	+	2.27 ± 0.001
*Rab7*	+	+	2.65 ± 0.001
*TUBA*	+	+	1.40 ± 0.001
*DVnein*	+	+	2.92 ± 0.001
*VAMP-3*	+	+	1.28 ± 0.001
*VATPase*	+	+	2.55 ± 0.001
*MHCI*	+	+	2.37 ± 0.001
*MHCII*	+	+	1.24 ± 0.001
*SRB1*	_	_	0.52 ± 0.001

### *F-actin, Rab7, TUBA*, and *DVnein* Gene Silencing Reduced the Replication of FAdV-4

**Figure 7:**
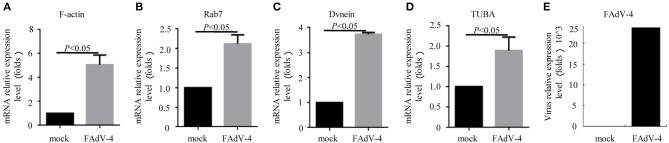
The mRNA levels of genes involved in the cell phagosome pathway in the LMH cell line, as revealed by qPCR. LMH cells were prepared in cell culture plates; one was the control, and the other was infected with 200 μL FAdV-4. The mRNA levels of **(A)** *F-actin*, **(B)** *Rab7*, **(C)** *DVnein*, and **(D)** *TUBA* were analyzed by qPCR, 24 h after transfection with FAdV-4 **(E)**. All data are presented as the mean ± SE of three independent experiments. Significance between the treatments was determined by the Student's *t*-test using the SPSS software (version 20.0). Label of *p* < 0.05 denotes significance between the treatments.

**Figure 8:**
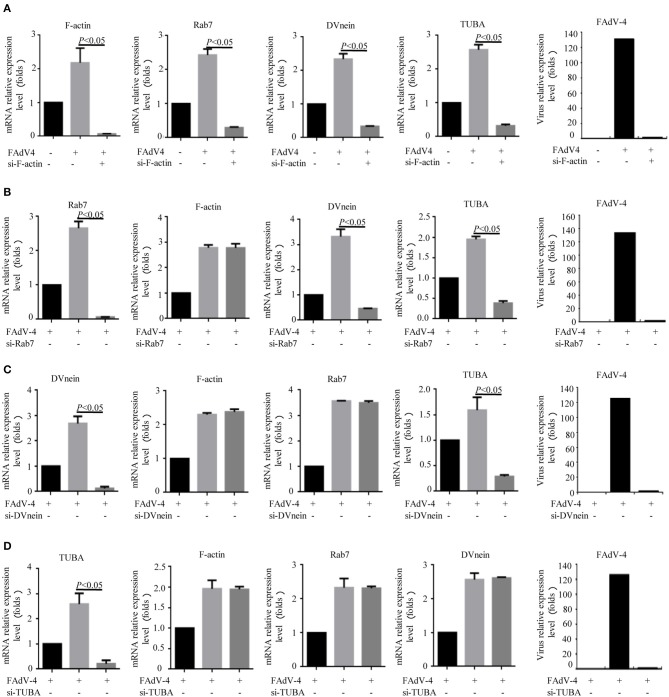
Knockdown of *F-actin, Rab7, DVnein*, and *TUBA* reduced replication of FAdV-4. LMH cells were cultured in 6-well-plates for 24 h. Three treatments were conducted. One was the control group, in which cells were siRNA and FAdV-4 free. The second, was the FAdV-4 infection group, in which cells were infected with FAdV-4 but siRNA free. The third group was transfected with **(A)** si-F-actin, **(B)** si-Rab7, **(C)** si-DVnein, and **(D)** si-TUBA, respectively, and 6 h after transfection, cells were infected with FAdV-4. At 24 h post-infection, all cells were collected. The mRNA levels of *F-actin, Rab7, DVnein*, and *TUBA* were determined by real-time fluorescent quantitative (fq)-PCR. All data are presented as the mean ± SE of three independent experiments. Significance between the treatments was determined by the Single factor analysis of variance and least-significant different multiple comparison using the SPSS software (version 20.0). Label of *p* < 0.05 denotes significance between the treatments.

Firstly, 24 h after LMH cells were infected with FAdV-4, the mRNA levels of *F-actin, Rab7, TUBA*, and *DVnein* were significantly up-regulated (Figures 7A–E). The four genes were then silenced by RNA interference. The results indicated that when *F-actin* was silenced, the mRNA levels of *Rab7, TUBA*, and *DVnein* were reduced, as the viral load declined (Figure 8A). When *Rab7* was silenced, the mRNA level of *F-actin* showed no decline, but the mRNA levels of *TUBA* and *DVnein* were both reduced, as the viral load declined (Figure 8B). In addition, we found that when *TUBA* was silenced, the mRNA levels of *DVnein* were reduced, as the viral load declined (Figure 8C). The viral load of FAdV-4 declined after silencing of the *DVnein* gene (Figure 8D). These results further indicated that genes of *F-actin, Rab7, TUBA*, and *DVnein* in KEGG pathway of phagosome was used to facilitate FAdV-4 invasion.

## Discussion

Hydropericardium syndrome (HPS) is an infectious viral disease in chickens, 3–5 weeks of age. The first outbreak was reported in Angara Goth, Pakistan in 1987 (Asthana et al., 2013). It is characterized by hydropericardium and hepatic necrosis. Reports indicated that broiler birds are more susceptible to infection (Dahiya et al., 2002). In the current study, we isolated a virulent strain of FAdV-4 from sick pre-laying hens, which also had typical hydropericardium and hepatic necrosis.

Virus would attach to target cells after they pass through the extracellular medium. F-actin reinforces the plasma membrane and imposes a barrier toward the outside of the cell (Wang I. H. et al., 2018). The F-actin participates the formation of endocytic pits and vesicles, which often is used by virus to bind to cell extensions (Mercer and Helenius, 2009). Then virus transmits forward signals into cells and prepares to endocytic uptake and infection (Greber, 2002). F-actin is required when the endocytic vesicles containing the virus separate from the plasma membrane (Liu et al., 2016). Furthermore, Rab7 mediates the process by which endocytic vesicles are transported to the lysosome (mature phagosome) (Suwandittakul et al., 2017). If the virus is released from, or is able to escape the lysosome, it would enter the nucleus. However, it requires the assistance of a motor protein and tubulin, such as TUBA and the DVnein protein (Leopold et al., 2000). Nevertheless, the detailed mechanisms by which FAdV-4 invasion and intracellular trafficking in host cells occur, require further investigation.

Transcriptome analysis provides a novel research tool that may reveal the interactions between host and virus (Wang et al., 2015). Researchers have revealed new insights into the host immune response to Muscovy duck reovirus (MDRV) infection using transcriptome analysis (Wang et al., 2015). They then elucidated the molecular mechanism of hepatic steatosis induced by MDRV (Wang Q. X. et al., 2018).

In the present study, our team found 13,576 DEGs that were screened in livers infected with FAdV-4. These DEGs were involved in “biological processes,” “cellular compounds,” and “molecular function.” The KEGG analysis revealed that the phagosome pathway was significantly up-regulated. The DEGs in that pathway were evaluated against by qPCR, and results supported the KEGG analysis. Then four genes as *F-actin, Rab7, TUBA*, and *DVnein* in phagosome pathway, were determined in LMH cells-infected with FAdV-4 by qPCR and RNAi, results also supported the KEGG analysis. The DEGs in phagosome pathway are involved in the formation of a phagocytic cup, intracellular trafficking along microtubules, and the internalization and formation of phagosomes. But how FAdV-4 regulates these genes to contribute its proliferation need to further investigation.

## Conclusion

In this study, the FAdV-4 strain NP was isolated from sick young hens. And transcriptome analysis revealed that the KEGG pathway of phagosome was used to facilitate FAdV-4 invasion.

## Data Availability Statement

The raw data generated during this study has been submitted to genbank, accession numbers MN604703 to MN604744, SRR10350642.

## Ethics Statement

The animal study was reviewed and approved by Research Ethics Committee of the College of Animal Science, Fujian Agriculture and Forestry University.

## Author Contributions

All authors listed have made a substantial, direct and intellectual contribution to the work, and approved it for publication.

### Conflict of Interest

The authors declare that the research was conducted in the absence of any commercial or financial relationships that could be construed as a potential conflict of interest.
