# Supplementary material for: Transcriptome Analysis Reveals New Insight of Fowl Adenovirus Serotype 4 Infection
Source: Front Microbiol. 2020 Feb 11;11:146. doi: 10.3389/fmicb.2020.00146 (PMC7026491; doi:10.3389/fmicb.2020.00146)
Supplement: Supplementary file 1 [file Data_Sheet_1.docx]

Supplemental data 1

1 TTCCGATCTC ATCATCTTAT ATAACCGCGT CTTTTGACAC ACTTACAACC GCCGCGCGCA

61 GCGCGTCGTG ATAGCCGAAA CCGAAACCGT TTAAGTGAGT CATCAATGAC TCATGGTCTC

121 CACCCAAATG ACGATTACGG ATATCCGTGA TGGACTTCCG TATTTGCGAA CGAACGCGAA

181 ATAATGACAC AGCTCTTGCC TCTCATTTCA ATCAGTTTAT TTCTGGTAAT ACGCGTAATC

241 TATTTTAAGT GGATCCGCAC ACCATTGCGT GGCTATGTTC GGATCGGATT GCGGTTGGTT

301 AGTCTGTTTT TCTTTCGCCG GCTGATATGA ACTTTCGAGC TTCTTCTCGG AGCCGCCTTA

361 TCTCGGGATC ACGCTGACGC TCCTCCTCGG ACTGGAATAT AGTGTGTTAG TATCTATCAG

421 GTAAGTAATG AGAGGGTGTG GGATCTGCAC TTACCAATGG GGGGATGTGG GAACCGCAGT

481 GAAATACTTC GGTTCCAAAG TTCTGCATTC CGATACGAAA GAATTCTGGT ATGGTGGTTG

541 CTAATTTATG GAGAGTGTCT TCTTCGTAGC CATAGACGGT TTTATCTTCC GCCACCAAAA

601 TGACTACTTG CAATCCATCC GCGAATGAGA TGACGTTGTC CACTATGCCA ATGACGGTCA

661 TTCTTTTTGG GGAAGGCAGA TACAGCACTT CGCAGTATTC CTTTAAATGT TGACCCGCAT

721 GTAAGGTACC TTCCAGATCT CCTATAACTA GAAAGTGTTT GCCGGACCCG ATGGGGGTCA

781 CTGTTCCGCG ATTAGCCTTA GCATACCTAT CTATTTCTTC TAGGGTACCC CAGTAGGGGA

841 ACCTGTCTAG CTTTACGGAG CTCTTCTCGC CTCCCATGTC GTGTTCTGTC TGAAATCTGT

901 TCATGTCAGA ATATATAGAG AAAGGAATGG GCGGACACCG AGTTGATTCA TGCTCTTATC

961 TTTATTGTAG TCACGCACTT TTATAGCGTT ATAAATTTTA CAACATTTCA GTAGTTTCCT

1021 GGTATACAGT ATTATTGCCT GTTCCAGGAA CAACAATAAA GGTTGGCCCT GGACCTCTCC

1081 CTCTCCTTCT AAGGGCAATG AATACGGCAA TGATAACACT GAGACAGACT AGGGCTAAGA

1141 GTCCAATGGT GACAGGAATG GTGACCGTGG AGTCTTGGTC CTTGTGGAGG GAATCTTTAG

1201 ATGATGGTTC CGCCCAGAGG AATTCTTGAA GAGGGGGGTT AGTACCTGGG GATACGGTGA

1261 GTTGAAGAGG GGGAGGTGTG GATGCTCTGC TGTTGCTTTC TTCGATCGGT TCCGTGCTGT

1321 TTTCTTCTTC CTCTATGATG GCAGTGCTGG AAGGAGTAGT AGCAGCAGTA GTTGGTTCTG

1381 AGGTTTCATT GTTCACTACT TTGAACGGGA GTTGTACCTT TTGAGGAACT TCTATCTGAT

1441 CGCGATATTT TTCGAACCAA CAATCTTCAT CTTCTTCTTC ATCACAAGCG GAGCTCGGTT

1501 CTGTGATTGA TTCGGTGGAG GACGTGGTCG TAGTGGGAGT AGTGGTAGTG GTGGTGGGAG

1561 TAGTGGTTGT AGTGGTGGGA GTAGTTGTAG TGGTTGTAGT GGTGGGAGTA GTGGTTGTAG

1621 TGGTGGGAGT AGTTGTAGTG GTTGTAGTAG TGGGAGTAGT GGTTGTAGGG GTAGTGGTAG

1681 TGGTGGTTGT AGTGGTGGTA GTGGTAGTGG GTTTAGGATG GACATTAATC TTGAGCAAGA

1741 CTTCTTCCCA TTGGTAAAAG AAGGAGATCC AGTAGGTCTT CGGGTCGGGG CAGGTTATGT

1801 TGAGAGTTCT CGTAGCTCGA TTTACTGTGA AGCTACAAGT GCTAGCTACA GGATCGCACA

1861 TGTCCCAGTA GGTCATGATG TTTTGAATGG TGGGCGAGGC AGGATCTCTC AGAGCAATAC

1921 GGATGAGTTC GAACGGCGAT GGCAAGCTGG TTACTGTAAC AGTCTGATCA GTCAAGACTT

1981 CATATGCGCC CATGTATGTA CGGAACATGT CGGTAAAGTT GGATTGTTCC TTCAGGCATC

2041 CATGTTTGGG AGGGACTTGG ATGTTGTGCC TTCCGTCACC CGTAAGATCG AGTTGCTGGC

2101 GGTACACGGG TAGGACAGCT TGCTTCCATG TTCTGTAGCA GCTCCACGTA TAAGTGGTTT

2161 GTTTCCTGCA ACTTAACATT TCGGACGAGA GGCCGGCTGT GGTGGGTCTA GGTAACTGCT

2221 GTTGGTAAGG TTTGGGAATC AGAAATCGGG CTAGATAGAC GGGACAGTGT TTGTAGTTCA

2281 TGTAGACCTC GAGGAAGTGC GTATCTGCCA TTCCGGGGGA GAGTGCGCTG AAGGATGTTA

2341 CTAAATCGTA TTTGTTTCCG TAAGGGATGA CACTGTAGTG GTAGCTCAGA TGGGAGCCGG

2401 CCGAGATGCA TCCCTTGTCG CTAATTGCCA TTGCCATGAA AGAGGGATCT ATGGAATCTT

2461 CGTGTTGGAA TTCGAAGGGT GAGAGCTGGT TTGCATCCGT GGTGAGTGTA CTAAGCCAGA

2521 TAGTTTCGTG TTTGAAATAG GCTGGATACT GGTAGTCTTT CCCTATGGTG TACCCATTCC

2581 AAACCGTATG CATGGCACCT TGATCTTGGG GAGGGTTCTC AGCATACCGG AATGCAGGTA

2641 GGCCTTGACG GGTGTCTAGG GACACCATGA AAAGAACAGG AGCCATGAGG TGTGAGCATG

2701 CATCCATGGT GTGTGGGATT ACACCCGATT TGGCCACCGT ACCTAAGTCG ATGTATTCCG

2761 TGACCTTTCT TCCTTGGTAA TTTTCGGCAG TGATAGTGCC TGTCCATTTG CCAATGAAGG

2821 GACAGTGATT CAAATAGGTG CCATCTATGT AAGGGGTAAT GTACTCATCC CCTTCTAATT

2881 CGTGGAGTCC CATCATCCGG CGGTTCGTCA TAAAGAGAGC TACATAGTCA GCATCTTTTT

2941 TAGACAGACG GGCTTTGGTG AGGTAAGGAT AGGGAGAGTT GGTTTTGAAG AGAGGTCCTG

3001 CTGGGTCGAG TCCAACAATT CTAGTACATT TTCTATTTTG GAGCTGGTTG AATCTTCGAC

3061 AAATTGCACC GCATGCATGA GCCCCCAAGG AGTGTCCTAT GCAGTGTAAG TTGGTGCGGT

3121 TTATGTTCTT GAGCAATCCG TCGATATTGA TAGTGACGGC GTGGTAAGCG GCATCTCCTA

3181 GAATGAGGTT ATCGGCGCCT TGTACCCCCC AATCGACTAA CAGCACGCCT ACGTCTGGGG

3241 TCATCTTCTG GTGGAAGCGG AGGAATTTGA AGAAGATATC AAAGGTGCGG TGGAGACCGT

3301 GCCATCCATG GACTAATAGG ATCAGGTTCT TTTCCCTTCC GCCTCTCAGG AATCCACGGT

3361 CACGGAGTCT CCAGTAGATC ATGTCATTGT CATGGAGAGG GCCATGGATT CTCGGGGGTG

3421 CCATGCCGTG TTGCCCATAC CAAAAGTAAT TGATGTGACC TTCATGGCGA ACCGGGCTGA

3481 AGTTGGCCAT GGGTCCTTTG GTAGGGACCG TGGGTTTGTC CTGTATGGAG TTGACGGCAG

3541 CATCCCCATT TAGGAACCAT GCAGTGAGGA GGGTTAGTAG GGCTAACAGC TGTGGATGGA

3601 GAGGTAAGGA GATTTATGTT AGCGAGGTTA GGTGAGAGAG TAAAAGGGGT AATGGATGAA

3661 TGATGCTTAC CCGCATGGTG TTTCTTCCTC AGGACTGGTC ACCGAAGAGA AGAGTATCTT

3721 CAGACTGGAT CAGTCTTCGG ATGGAATACC GTGGTGCGAG TCTGCGGAAA GTTTATAGGG

3781 ATTTCTGGTT AATGATTGTC TGATGCTGAC GTGACCGAAA CCGCTGATTC AGTATCATTA

3841 TGGGTGACTC ATGTACCTTG TGATACCTTA TCTAGGTAAT GAGGAAGTAC TTTGTGATCC

3901 TTATCTAGGT AATGAGGGAG TGACCTTTGA CCGGTGATTC ATTCTGGGTG GGGTTGGAAA

3961 TTTTCCATGA CGTCTATGGT TAATGATTAA AGAGGTAGAC TGTATAAGTA CTACAGAGTT

4021 TGGAGGACTG CAGAGCGCTC ATTTGACAGC CCAGTGTGCG GTGGGTAATG TTGGGTTACT

4081 TAATGTTGCG CGTGCCCTTA TGGGAATTGA ACCTGGCTAT GTTTCACGAT TTTCGGCGTG

4141 ATATAATTAA TGCTTGGGAG AAATTGGATT TAGGAAAAGT GTTTCCTGGA CTAGTAACGG

4201 GTTTCTTTTA TCTCTATGCT TTGCAAGATG GAGGATTTTG TATCGATTGT TTTTTGCCTT

4261 GTTCGGATTT TGGAAGCGCG TATCTGGAGG GGGTTGGTTT GGCTGTTAAG TGTTTGTTTT

4321 TTGGGAAATA TCCATATGAC AGTGAGAAGT GGATTAAGGG TGTATGCCTT TTGCGTAACG

4381 AGGGATACGC GCAAGAGTAC CGCTTGGGTG AATATTGCAT AACATCAGAA GCGGTTGGCA

4441 GTAGAGAGGG ATGTATTTAC GAGTGCGTGT ATGAATAAAT GGTTTAAATT TCAGATGACA

4501 ATGTACCTTG TGTTCGGAGC GGCAGTTCCG GAATGGTGTC AGAGGCCTGA GGTTTTCAGA

4561 TATGTTCATC GCGGATTGAA GTTCATATGG ACTAAACTTG TTGAGAAGTA CAGTCCCGAA

4621 TGCAGTCCTC TGAAGTACAT GTTCGGGTTG CAAGAGTTTT CCGTGTGCGA ATGTCATGCA

4681 ACTTTGTTAA TTTGTATCCA TTGTTCCGAT CAGACTAAGG GAAAGTTGGA GAAGTGTCGG

4741 AGTGATATGC GACGGTTCCT GCATTTGGTT TTTTATAGAG ATACCGGCTC TCCGTGCCAG

4801 GTGTGGTTGA AGCTCTATGA TAATCAGTGG TGTCCCGAGC AGTACCGCAT GGGGCGCTGG

4861 GGCGTGACGG AGGAAGCATT TCAATGTCTT GGGGCGTGGC GTGTATGTAC TGGACTTCGT

4921 ACCGCTGATG ATGTAAATGT TGACTGAGTT GTTAATAATT AAATTTGATT ACAGTCTCTT

4981 CCTTTGTGTC ATGTGATTCA GTTGGTTAAT GATTATAGCG GTGAAAAATT TTTCTAAGTC

5041 CGGAGCTCCG CCTCCGGACA AAGTTCGAGT TAAAGTTTAA CTTTAGAACC TCCCCTTTGC

5101 GCACGTCAGT GCCAAAGTCC GTTGCTCAGT TAATGATTGA TAGAGTGGAA AATTTTCTAA

5161 GTCCGAAGCT CCGCCTCTGG GCAAAGTCCG AGTTGAAGAT TAACGTTAGC TCCTCCCCCT

5221 CTCATACGTG GGGTTCAAAG TCCATCGTCA AGTTAATGAT TGATAGAGCG AAAAATTTTC

5281 TAAGTCCGAA GCTCCTCCTC CTCTGACGTA GCAGTCATGT GACTCAAAAG GACAAAGTCC

5341 ATCGGGGCGG AGAGAGAGGT GGAGCTATAG GCGGAGTTAA AGGCGGAGCA TAGTGACGTA

5401 GGCCCATTGG TAAATGATTA AGCAGGTGAA AATTTTTCTA AGTCCGAAAA GTTATTCAGT

5461 AACTAAGTCT CCGCCTCATA GGCGGAGTCT CTATTTCTAT TGGTGTTCCC CAGGACCGCC

5521 CACAAAGAGG AGGAGTCGTA TTTCTGTTTT CTGATTGGTT TATTAATATG GCATGAACCG

5581 TAGCCCCGCC CTTTTCATTT TTAGACAATT TTCCAACACA CCCATTTTCG CGAGGTTTTG

5641 CAGAGAAACA GGGTTCTTAG GACCCCCATA GGGACCCTCG GTAGGGACCA CCACTCGCCA

5701 TTTTTCTTCA CCGGCCGAAT ATTTGAAAGT CTTATAGGCC CAAACCGAGG GTTTTTCGTT

5761 AAAAAATCGG GAAAAGATGG GTCTAGGAAT ATGCTTTTTT AGATCGGTGG TCGTCAGGAC

5821 CCCCTGGAGC ATATCTTCAA AATGTTGGGG GGCTTCTACA TGGACCTCCT TGGATACCGG

5881 TATAGGCATC TCCCCCTGGC GGTTGAATGG AGTATTGACA CGGAGGGTCT TTAGCACCAT

5941 CTTCAGGCTG ATCTCGGTAG TGACGGGGAC CCCCCAGCCC CAGGTGTGTA TACCAGTATT

6001 GTGCAGTAGG GGGCTCCCGA GTATTTCATC CGGCAGATCC CACAGCCCAT TGGCTACGGC

6061 CTCCATGGTC AGCTGGGCCT CACTTCTCTC CCATTGGTGG ATACTGCATA TCCGGGCCGC

6121 CATATTGTAG AGGCGGTACT GCTTTCCCTT ATGGAGGAGT CTCCGCCCCT TCACGAGCCA

6181 CGTGGTGAAC TCCAATGCAT TGGATTGGTC GGGAGTGAGG GCGGGACTGA ACTGAAGGGC

6241 CTCAGCCAAT TGGTTGTCGA GGTACGGGCG CCTCCCATCT TCTCCGCCCC TCGGCATGGG

6301 ATCCAATAGG GTGAAGGATA TGGTAGCCAC CTCATAGGGC CAGCGGGGAT CATGTGATGT

6361 CAATGGATGG GATCCCATTG GCTGTAATGG TACAGGGGGG TGGTCATGGG GGGTGGTCGG

6421 CTGTACAGGG GGGTGATCAT AGGGGGTGGT CGCGTGTACA GGGGGGTGAT CATAGGGGGT

6481 GGTCGCCTGT ACAGGGGGGT GTACAGGGGG GTGCCAAGGG GGGTGGGAGT TTGATTGAAG

6541 AAAATCACCA TTAATTTCAA TGGAAAATCA TAATATTGAG TTGATTCGGT ATTTATTCAT

6601 CGATTCAATA TAGAGGCCTA CCGGTTTAGT GGAGAGCTCC GTTCAGTCTG CTGGTCCCCG

6661 GTCTGTCTGT CTGAACCTGC CTACCTGGTC TACCTATATA GGTTGAACGG GCTAATGTGT

6721 AACTACATGA CATCATTGAG AGCCAATGGG AGAGAGCTCC GCCTATCACG CCATCGTTAA

6781 TGTATAACGG AGGAGGCGGA GTCGGATGTC CATATATGGT ATGTTGCCAT GGTGAATAAA

6841 CAAACAGGAC AAGGTCCATG TTAATGGTTA ACATGGAAAA TTCCACTGGT GTCAGCAGTT

6901 TAATGTTTAA CATCACCATG GTTACATCTC CATGGCAACA CCGACGTCAC CGTGACGTCA

6961 ATCGTACGTC ACTAGCTACT TATTAACTTA GAGTTATTTA TTAACTATAG TTAATGGTTA

7021 AACCATGTTA ATGGTTAACC CATGTTAATC ATTAGCCGAG TTACATATTA ACCAGAGTTA

7081 ATCATTGACT CAAGGTTATC CATTAACTCG GATGAATCAT TAACTTCAGC TTAATATTTA

7141 ACTAGCGTTT AATATTTAAC TAGAGTTTAA TAATTAACTA GAGTTTAATA TTTAACTTGA

7201 TTAATGATTA ACCGCACTTA GAATTTTTCA GGTTAGAAAA ATCTTACTCA GAAAGTTTTT

7261 AATGTAAAAT TTATTTATTG TGGTTGATAG TCAGTCAGAG GCATGACTTT TGATGCGCGA

7321 TCCAGGGCAG TGTCCATGCA CCACTCCATT ATTCCTCCTT TTAGGTCATT GAGCCACCGG

7381 AATCCCAAGT CCAACATGTC ATTGAGGGGT TCGCAATGTT GGTAGTTGGT TTCGTCTAGT

7441 AAGAGTTCAT TGAGATGCTC TTTGGCATCT TCGGAAACGG CGTGCATTCT CACCAGTGAG

7501 CAGTATGCTT CCTGAAATTT CCTGTCGTTT TGCATGCACT CGAGCGAAGC TACGGTTTTC

7561 AAGTATGCGC CGTGCGATTG CCAAAACTCA CGGAGTCGGG AAAAGACAAT GGTATAGTCT

7621 AAGTCATACA AGTCGACACC GGAGAAAAGT ACTGAATCCA AACTGGAGGA AGAGTCGGAA

7681 GGGCTGGTCG GAGGTCCCTC TATCGGAATA CCGAGTTCGG CCATGATGTC TCCTACCTCT

7741 ATGGTTTCGA TCGGCGGTGT TTCCTGGTTC TCTTCCGGAG AGTAGTCCGG GGGACCGGCA

7801 GCCGCCAGGA GGTCATCAAA AGACAGCATG GAAATATATT GAAGAGGGGC ATGGCACTCG

7861 GTCTCGAGGG ACATGTCCTC ACCTTCTCCT TCTGGATTCG GCGAAGTGGA GGGGTGGAAA

7921 AGGTCGAGCC ACTCTTCGGC CATGGTTCCG CAGAGCCCTC GTGTTGGAAG TAAGGTTGCT

7981 TTTCGCTGCG GGCTGGAGCC GTCCTTTTAA GGTTTACTGG CGATGACTCA TCATGGGTGT

8041 GGCGTTAATG ATTAACTTTT GGTTTCGCTT TGTTAGCAAG TGGAAAGTTC CATTGTTGAC

8101 TTAGCGATGA CTCATTGATG ACTCATTGTC AGCCACCTGC TGACACGGCA ATTAAGCGGG

8161 TCAATGAGGA AGTTAGTGTC TGTTAATATT TTACGAAAGC TGTGTTTATA TGGCGGATGT

8221 TCGGATCGGA GTTTATTCGC CCGGCTAGTA CTCCACTCGA GAAGGAGCCT CTGAGCCGTA

8281 CTCTATGCAT TGCGTGATTG TGGGTGTCGT CAGTGACGGG ATTAGTATAC GGGCCACCCC

8341 TGATTCTTAT GTTATCCCGT GATATTAGGT CGCGCATTCC GATAGGCTGT GCCTGAGCCA

8401 CGAAATGACC TGCTGTTATT TATTTGTTCT TCTAGACCGT GCTGTAAATA AAATCCTCGG

8461 TCTGCTGTAA GATCATGCCT CTGTGTGCTG AATCGGTGAG TTCGGTTTCG GGTTCGGGGA

8521 GGGTGATTCT GTTGAGAGTT CTGAGTAGAG CTTATTACTA TTTCTAGGAC GTTCTGGCCC

8581 TGGTTCGCGG ATGCGGTTTT GAAATGGAAT GCGGGTGTTG TTCGTTCAGT GTATTGGTAC

8641 CCCCACCTCG GGAGCCTATC GTGCTCCATC AGGAAGAGAT TGAGAGGATG ATAGAATATC

8701 ACCTGACTTT GGCGATATTG GATCTGAACA CTTTTAACGG AGATGAGTTT GTGCGCTACA

8761 TACATTCATC GATTTACGTA GCGGTGGGAT GTAGGTGTTC ACGCTATCTT CGCCTGCGGT

8821 CCGGAGTTCA CTTAGTGGTG AACTGCGATA TACGCTTTCA GACCGCTATT CCGCTCACCG

8881 CTTCTGACAA GCGGGAATTT TTGCAGTTCA TTTCCCGACG TCTTTTTAAC GTTCCGCCTG

8941 AGATAAGGGC TCCCATGGTC AGGTGGATGG AAAGGAAAAG GAAGACCGAT TTTATTTTCG

9001 TACCGAGCGG ATTGGTTTTG GGGAGCATGG CATGCTGCAA GATGGCCGTG CGCACCATGT

9061 GTTTTGAGAT TTCCACCTCT AAAGTTCCGC GCTGTGTTCC CTTTATGGAG GTGCCCATCA

9121 ATTACCTAGA ATGTTGCAAA ACCAATGTGA GGATGACTCT GGTCTGTCCT CCAGGTAATG

9181 GTAGCTGTAT CACTCAATCG ATATGCACGA AAAGCATGCT GGTCGATTTT GTGCCATTAC

9241 CGTACAGGGA AATCGTGTTT CGCGGGGTGT GCTATTTACC AGCTCACAGG CGCGCCACCT

9301 ATAGCGACAT GGAAGAGTGG TTTATGCATG TGCACGGTCC CTTCTGTGAT TGCGAGAAAG

9361 GGTGTGATCG GTGCGAGGTC AAGTCTCCTA TGAACTTGTT CTATCTGGCG CAAATGGCTT

9421 GTTTGAAGTT GGCCTTTGAC CGCAGACGCG CTCGCGTGAC TCACAATCGC AAATCTCCGT

9481 TTTTTAAATA GGAGTGCCGA CGGAGATATC TCCCGCTGCT GCTGATCGGC TTTCTGACGG

9541 TCGGTCTCTA CTTTACCTGC ATTTCCGTCA GTTTTCTTTT CGTGGAACGC TCCGTCAGTC

9601 TGTTGCTCTA TGTCATAACC GTGTTTCTCA CCAGCAGTTT AATGGTGTTG ATTGTGCTGT

9661 ACTTTATTAT CAGGGAGTGG ACTTGTGAGG TTCCGACGGC CCGTTGCACG CCCCCAGAGG

9721 TCCGGTCCCT CAGAAGGCGG GGATCGGGTA GGTGGGTACT GTCAGCCAAT CCGCTGTACT

9781 CTAGTTTGCG CATCGGGTTA GGCGCGTCGC GACCGCTGCC GCCTGTGCCT GTCAACCCAT

9841 ACTCTGAACT CGAGGACCCT TACGATCACG TGTACACTTC CATTTCGCCC TACGAGACCC

9901 AGGTTTAGAG ATGGAGGAGC GTGACGCATC ACCCGAACCG ATGGAGGAGG CCCCCCTTGC

9961 CGAAGAGGAG GAGGAGCTCG CAGCGGCGGA GGCCGCAGCC AATCCGCATG AAAACATGGT

10021 GCTTGCTTGC ATGGAGAGAA TGAGGGTGGT GGTCAAAAAC TGTGTGTGTC ACAATTACGG

10081 TTTGCGCAAT TTGGGGTTGT CTGTGGGAAC CGGGGTGTAC TGTAGGTACG GGGATAAGCT

10141 GTGCGAGGGA CTTTCGGAGG ACTACGGGGT AGCCGGGAAC TATTTTGTGT GCGCGTGGGC

10201 GTGTGCCATG TTTTCCTCCT TTGGACCCAT GGTAGTCCAC CACCTGCAGG GCGTGGTGGG

10261 CATGATGATT CATGTACCTC TTTACCCTAT CGACAGACGG GCCGAATTCC TGATGGTAGT

10321 ATCCCAGCTT GGAGCCTTGG GGGCGCTCCC CTGTCACTAC TTGCGCAAAA TGGAAATCAG

10381 TGTGGAACAC AACATTACTC GCTTTTACGC ACCCGATTGG TTTTTAGAAA GCGTAAATCT

10441 GTTGTATGAT TGGACGCGGG AACAAAGGAG AGAAGCTTTC GGACAAATGG ACGATCAATA

10501 AAAGCAATCA ACGTTCATGA CTCTTTATTT GACACGCGGT GGGGAGGGCG CGCTTACGGG

10561 AGGGAGGCCG CTGGACAGCT GTAGAGCACG GGTCCCACGA TCATGGTTCC GCTGTTGTTT

10621 GGATTAAAAA TGCTCGCGTT CGTGCACTGC AGACTATAGC ATAGAAGGGT GTATCGCTCT

10681 CCGGAGGCCG AAACCGGCAC GGGGAGGACG CGGATCCCTA TGTTTCCCGG GTTCCAGGCA

10741 CCTGTTACCA CCGGGCTGAA CACTTGGAAT TCCCCGATGG ATGGTTCATA GTATCCATTG

10801 GCCGAGTACG TCCATGGGCT GGTCACGCTC CTATTGGCCA TGGGTTCAAA GTCCGTGAGG

10861 GTGGCGGTGG AGGGGCTGAC CGTTCCCGCT TGAATCCCGG AGGGGTTGCA TTGCTGGAGA

10921 TAGGCGGACA CCCAAAAGGT GAACCATTTG GCATTGGCGG AGTTGAGGTC CCCAGGGCGA

10981 TTCCCCATGG TGGCGCTGTC CAATTTCAAG TAGAGGGAGG TAACAAGGAG CCCCTGTATG

11041 TTCCACTGTT GAAGGTAGTA GGCGCAAGAG AAGGCGTTCG CGCTGGAATT GACGGTCGTG

11101 GCATTGTAGG TGTTGAGGCT GGGACTTCCC GAGACAAAAG TAGCGATGGG TGTGGAGACG

11161 CTCCCCCCTC CGACCACGGT TAAAGCGCCC GCGGTCACCG CTAGGGTATT AGTGTCGTAC

11221 TTCAGCCCTA GGCCATTGGC GGAGACCGTA AGCGGTCCGC TGGGATCCGG TTTCACTTCC

11281 AGAGTGTTGT TGACAATCTG TAGGCTTTCA TCCACGGAAA CGCCCACTCC CGAACTGTCG

11341 GCTTGGATGC CTCCCTGCGC TTTTAGGTTG AGTTCCAGCA CTCCGCTTCC GGTCGAGGTG

11401 TTGACCGTGA ACATGTTAGG ATTGATCTCG AGGTCGATAC CACTGCTATC GGCAGTGATG

11461 GGTCCTTGTT GGTTGAGGTG TACGCCCAGT TCTCCCTGAT CCACGAGCAA GGTGTCGTCC

11521 ACGCTGACCC CCAGTCCACC CGCGGTGGAA TCCAATCCGC CGGACGGGTC GACTTTTACG

11581 GCCAGTTCCC AGTCATCGTT GACCATTACG GTCACTCCGT CGACCTTGAC GTCCAGTCCA

11641 TCGGGGGTGA TGTCCAGGGC CCCTTCGGGG TCAACGGCCA CCGCCAGTTG ACCTTGGGCG

11701 TTGACATCGA GACTGGGGTC GTGGGCCAAG TCCACCGATC TGTTCTTGAT GATGATGGGA

11761 TCGGTGACGT TGAGCGTAAG CTGTCCGCCC TGGTCCACTA GGGGTCCTGA GCCTCCCAAA

11821 AAAGGCGGGT TGAGCCCTCC GACGGGGTCG GCCACGTAAT CGAAAGGATA AACCAGGTCA

11881 AGCTGGGATG CTCTCACCAT GCGTTTGGCG CGCTTGATTG GAGCCGGGGA AGGTCCCGCT

11941 TCGGTCTCGG GCTTCCCGTT TTCGGAATGT CTTCTTTTAG GGGCCCGGAG CATTGTTCCC

12001 GTTGGGGGAG TAGACGTAAC CCTGATAGGA AAAAGGGATA GGACCGGTCG TCACCACAGT

12061 GTCGGGCGCG TTCTGGTCAA ACCAGTTGGA GCCGGAGGTC TGCGGCAGCG AGACAGTGAA

12121 GGCTATCGCA TCCGACGGCG TGGTTCCGCT GGACGTGTTC TGGATGGTGG CGGCGCAGTA

12181 TCCCAACGTG CCCTGTCCGA ATTTCTCGTT TCCGGTGGCG GTGGGCATGA AAGAGACGAG

12241 CCGCAGGCCG GGCGAGTCGA TGGGCACGTA CCAATTGTCC CTGGTATGAG GCGGCAGCCC

12301 GAGATAGCCC ACGTCGCTCT GGTTGCTATT GGGCACAAAC ACGTGGTTTT GATTGGTCGG

12361 CGAGACCGTG GGCGGCACGA TGAGGGACAA ATTCACCTCT GTTTCTATCG GGTACATGGG

12421 GCTGAGCACA AAGGTAAAGT TGAGACCGCT AGTCAGGCTG TTTTCTCCGC TCGCGCCTGT

12481 GAGGTCATGG GCTAGCTCCA GAGTGATGAG TCCGTTGACT AGCCCGGCTG AGCTCACCAT

12541 GTAGATGTAA TAGCCTATGG ACCAGTTTTG CAAGCCCTTG CTTTTTACGT TGCCGTTCCT

12601 CTGCGATATT CCCAAGACGG GCGTGACTTC GTAGGTCGCA AAGGGGCTCA GATAGGTCGG

12661 ACCGACCGAG GAGGCTAGCG CTAAGACGCC ATTGTTGACA ACGAAGTCTT GAGTGTCGAA

12721 CTGGAGAGTG ATGCCTCCCG TGTCGGCTTG TAGAGGTCCC TGTGAGTCGA CGAGCACTTT

12781 GAGCACCCCG GTGACGGAAT CTAGCGCCAT GGTGCGAGGG TCGAAGCCTA GCGTGACGCC

12841 TCCACCAGTG CCCGAGTTCA GAGGGGCCGC GGTTTTGACC ATGAGCGCCC CGTTGGCTTG

12901 CTTAGTGGCC AAAGTATTGT CATCGATGAG CAGACTGACC GCTCGATCGC TGACGTAAAT

12961 GGGCGTTTTG GTGTTGAGGG TCAGTAGGTC ACCAGTGAGT TCGAGCGGCC CATCGGGGTC

13021 GACCGCGATC TGCTGACCTC CACCTCCCCC ACCGCCGCCA CCGCTGCCAC CTCCTCCCCC

13081 CGCATTGAAG TCAAATGGAT ACACCAGATC GAGGAGGGGA CCATTCGCGC GGGTGGCCGG

13141 TCGCGCGCGT TTGGGTTTTC GACGCTCGTC GCCCTCCGAA AGGTACCGGC TAGGTTCGGA

13201 TAGGGCCCTG CGGGGTCGTT TTTTTCCGCT GGCGGAGACG GTATCGGCTG CGGAGGCGAT

13261 TAGGGCCGAC ATGGCGGAGC CTAGAAGATA TTGGTTAAAA ATAAACGATC AGGCTATGTT

13321 ACGGTTCGAC GAACCGCTTA ACCCTGGTTT CGTGTTTTGG TTGAGACGCA AGTTTAGAGC

13381 GCGCGTACAC AGCGAAGGCG GCGAGCGGGT CGTAATGACC AGAAAGGAGC CATTTTCCGC

13441 TTCGGAAATG CAAGAGCTTT ATTCCGAGAC CGATTACCGT CAGCAGCGCG TAAGTACCGC

13501 CCTTAACCCT GCCAGAACAC CGGTTCGAAA GCGTTGGTGC CCTTGTAGAC GCTGCTGAAG

13561 TAGGCCGGGA AGGCGTCGGG AGATTCGCTG TCGAAAGGGT GGGGGACCAT GGCCGGGAGG

13621 AACGTGGTCA TGAATTGGCG CTGGGACATG ACTCCGGGCT CGTCCACCTC CTGGCTGGGA

13681 CCTTGAACAC GTAGATAGTA CTTGAACGCG TCCGGGGTCA TTTCGGGGTA ATCGGCCGGG

13741 AGACCGTTGC CGGTCAGGGT GGTGCCGCAC CATCTAGGCG GTCGTAAAGT AGGGCGCGCC

13801 CGCGTCGGCA GTTTCCCGGC TGCGCCGCCC GCTAATTGCA TGCGACCTTC GGTCAGTCCG

13861 CCCGATAATT GCACCCTGCC CTCGGTGCGA CCTCCCATCA TGCCTCCGGA GAGTTGTACC

13921 CTTTGGACTC CGCTGGGGTC AAATGGGTAC ACCGCGCTGG GCGGCGGCCC GCTTCGCACG

13981 ACATCTGCCG TGATAATGTT GGTTTCGTGC GGACCCGCGT AGGGCTGTTG GTCTGACTCA

14041 GCCTCGAAGC GCGCGGTAAT TGCTCTCGTG ACCGACGGTT CTGGGAATCG GCGCCTTATC

14101 TCATCGGCCG CGTAAGCGAA ACTGTTACCG CCCGGCAACA CCCAGTCGAT AGTGGCGCCG

14161 TAGTTCTGTT GGGCGCCGGC GCATTTACCG GTCACGGGGT TGTATTTCCA CACGTAAGGG

14221 GTGGGTGCGG CGTTCAAGAG GTTCATTTCG ACAGTATAAA TGTCCCCGGT CTAGGCACTG

14281 CCACTCCAGG CGACTAGGAT TTTTTCCGCG TCCGCCTTGA GCCTGGTGAG TGCTTCCTCA

14341 GAGCTGGTGT AATGCACTTG TCGAGTGATG CTCTCCCGGG TGCGATTGCG CACGGAAACT

14401 CGCCGTTCGG GGTTTTTTCG AGCGTACTGT TCGATCTTGA GCAGGATCTC GAGAACCTCG

14461 GCGCGAAGGT CCGCGATGTG CTGGGGTATC GGTTTTTCTA GAATCGAAGA AGTGGCCGAA

14521 GCGGGTTTTC TAGGTTTAGG CGGCGCAGCA GGCTTTCGGC TAGCGTTGCG AGCCCTCGCA

14581 GCTGTGGGTG AAAGCAGAGT CGTCGGTCAT AGCTAGTAGT TTTCTAGCGT AGTAAGCCAG

14641 CACGGAGGCG GGCACGTACA GCTGACGTGT ATTTTTCAGC ACTTGGGCTG CCTGAACCCT

14701 ATCGAACACC GTATCCCGCA GCGCCTGACA AATGGCCACC CGATAGCGCG CCCAGCTCCG

14761 GTAGTTACCT CGGGGTGCTC GGTCGGGTAG GCGCTGCGGA CGGATCCTCC TGGCGGTGCT

14821 GCTCGTGTTG GCGGTGTTGT TGGTACGCAG GGGAGGTCTC GTCAGGGCCT GGGACGGGGC

14881 GACGGAGGAT GCGTTTCGGC TGCGTTTGGG GGGAATCGTA GGGGGGCTGT TCTCCTTGTC

14941 TTCCCCAGCC AGATCCGCGG AATACGCCTC GTCTTCCTCC TCCTCCTCCT CTTCGGAGAT

15001 CGTGCTCATC TGGTCCTCTT CCGTATCGGT ATCCTCGCTG CCTAGAGTTT CGCCGTCGGA

15061 CTCGGTCGCC TCCTCGGGAT TGTAGATTTC GGGCTCCGGA GGTTCCGAGG CCCTGGCCTT

15121 CAGGTCGACC ATTCTCTGGG CCATGAGGAG TTGCTCCTGC GACGGAGGGT TGGACCGAGC

15181 CGATAGCGCG CTGAAGAGCA CCCCCGTGTT CGGTTTCGCC GGCGGCGGTT GCGACGCTGG

15241 TAGGCCGTAG AGCTCGAGAA GGCTCTGCCG ACGTCGCAGC TCCTCTGGCG TGATCGGCGT

15301 AAGCGGTTGG TAGAGCTTGT CGCCGGGCTG CTCCAGCTTG GAGATCTGGC TGCGGAGTGA

15361 GGACCTCGCC GGTGTCGGGG TCTTTGTATA CGCCCTTACC CCGCGTGAGG AGGAACTCCT

15421 CCCTCGAAGC CTGAATTTGA CGAATCAGAC TGAGGATTTC GGGGCTTTTG GTGACGCAGG

15481 CCGTGAGGTC TCTAGAGAAC GCTTCCTCGT GTTGAGGGAA GTGCACCACT TCGAAAGGGT

15541 GGTAGTCTTT GGGTTCGAAT TTGTCTAGGT AGGCGTTGGC CCACAGTTCG GGAGTCAATT

15601 TGAAGGTCTT GCCGTCGTCC GTGCGGATTT CAAACGTGTT GATGGCGAGC AGTTCGTTGT

15661 GCAGAGGCAC GTTGTGTTGG GGCATCCGGT GCGGACTGCA CAAGTTGCAC GGACAGTAGG

15721 TGCGATCGCG AGGGTTGGGA TTCTCGGTTT CCTCGGGCTC CCACAGGTAT CCTCCGTGGT

15781 TGCAGAGGAA AAAGGCGGTT TGGAGGAGGT ACACCTGGTC CCAGAGCAGA GGCTGGGCTT

15841 GTCGGAAGGA GAGCGGCACA AAGTCGGAGG GTAGGAAGGG GGCGGCCGTG GTGGGAATGT

15901 TACTGCGCTC CATGATAAAC TGCCTAAAGG CGCTGATTTG GCTGCCATTG GTGAAGTTGG

15961 GGAGAGCTTT GCGCATTTCG GCGCACAGTC GGTCTCCGTC CATGAGCACG TCTACGATGG

16021 CTTTGGAGAT CTCGGTGGGT GTTTCGAGAG CGTAAATAGC TCTTCGGAGC CGAGTAAAGG

16081 CCTCCCGATA AGCCTCGATG GTCTCCTCCT GGATGGCTTG CTGCCACATG CCCATAGCCG

16141 TTTGCCACGT GAGCACCAAG AAGAGGTAGA TGCTGTCCAC CACGTAATCT CGCTTGTCCG

16201 AGCCTTCCAT CAACTTGGCT AGCATGCAGT TGTTGAGCGG GTCGTTGTAC GTGATCCCGT

16261 GGAAGGTGGC ATAGTTGCTT AGATTGACTT TGGCGGTTTC CCGAATGAGC GCCACGAAAC

16321 CGTGGTGGAA GGTGTGATGG AGCACTTCTT GTGCCTTTTT GATCGAGGAA GGCTCGCGGA

16381 ATACGCGTTC CATAAGCTCT AGCTGAGCGG TATAGCGGAC GGCCTGCGCT ACCATGCGCC

16441 GTCTGACCTC TATCGCTCTA GCTAGCGCGT GTGGTTGCAT CACGCCCTCC GGATCGACGA

16501 TGCACGCCAG TTCCGCGTCG GAAACGACGG GCTCGGGGTC TTCTTGCTCG CCACCGCTCG

16561 CTACCTCTTG AGCGAATGGC TGGAGCAGTA CCTCCATGAG CATGCGGGAT ATTTTGGGAG

16621 GCATGTGGAG GGAGGGGTAG CAAAAATAGC GTACGTGTTC GCCGCGCATT TTGGCCCACT

16681 CGAGACGGGA GGTGTCATCG CGCAACGGCA CTAATTTGGC GTCTTTCAGC TCTGTTATAG

16741 GCGACACCTC GTCTCCCAGA CCGTCGTCGA CCGTCACGCC CTGCTTCCAG CGAGGTAAGC

16801 GTTTGAAGAA GCGAGCGTTC TCCGCGGCAC GGAGCACCTC GGATCCGCTC CGGTTGGCCT

16861 TGCAGCTAGG CGGGATGGGG GCGGTTACCG CAAAGATGTG GTAGTTTGCG ATGGCTTTAG

16921 GCGTCATGAA GGGCGGATAA AAGTTGTAGC GAGCCTCTAC GTGCTCGCGC GGCACTTTCG

16981 GGTCGGGGTT GAAGAGAAAG CGCTCTAACT GTCTCTGCAC CGCGTCTACG GTAAGTGGCA

17041 TGCTGTCGAG GGGCGGGTCG AGCTGCGCGT CTTTGAGGGC TCCCGTTAGG ATCATGGCTT

17101 GCCTTTCGAT GCACTTGCGC AGCCCTGCAG CAAACGGATC GCGAGTCTCA GTAGCACAGC

17161 CGAGCTCAGA GTCGCTAGAG TGGCTAAAGC AGCCACCGCG ATCGGGGCTA GACTCGCCCG

17221 ATGAGCGTCG CACTGCTGTG GATGGAGTAG TCGGCGCGCT AGACTCGTCC GTAGGGGTGT

17281 CGGGTCGAGC GGCCTCTCCG TCGTAGCCTG CATCGCGTCC CCCATCTGCC GAATAGTAAC

17341 CGCTATCCGC GTCCGATTCG CCCGATCGGT CGTCTCTGTC GGCCGGTTCA TCCGGTTCCC

17401 CATCGTCCGG ATGCTCGTCT TCCGGCGCGG GGAACTCGCC GTCGGCGCCG GCGGTGTCGC

17461 TGGCCTCGCG CTCAGCTGCG GGCTCCTCGC CACGGGCTTT ATCCCCGTCG GCGGTGCTTT

17521 CCATCTCGCC CACGTCTTTT CGGACGTCAG CCTCGGGGCT TTTGTTCTCC TCGGGAACAA

17581 TGGAGGGAAG GTCCAGCGGG AGTTTAGGTA CTTTCCCACT CGGCGCCTCC GCTTCCTCTT

17641 TTGGCGCTTC CTTATCGCGT TCGATAAGGT CTTCCAAGAC TTTGTAAGCG CTAAGCACCG

17701 CCGTTTCTTG GCCCAGTCGG TCAATGAGTA TCTCGGCCAG GGTGTCCAGC TCGATCTCGA

17761 CTAACTCCTC GCTAGAGGGC AGCTCTTTGC GATAGGTGGT ACTAACGTGG TCGCGAAGAT

17821 GACTTACGTA ATCGGTCCAC GGAAACCGCT CGAGGGCGCT GCGCAGCACT AAGCCCACTG

17881 TCGCCAAGCT TTTCAGCTGT CCGTAACCCG ACGGATTCGA CATCATGATT ATGTGCGCCT

17941 TAGTCCCCTA GAGAGCAATG GAAGTCGAGA TAGATTCAAG CCGAAGCGAG CACAGCGACT

18001 ACGAGGACCC GATGCCCTCC GACGCGGAAG AGCAGGAGGA GCGCGAGAGA AGCGCGAGAC

18061 TCTCGACGAG CCGGTCGTAC GGCTCACCGA CCAAGAGGAA AAAGAAGCTC CAGAGGGACG

18121 CGGCCTACAG AGAGCCGCTA ACGAAGACGT TCAGCTCGGA GGACGAGAGG GAAGCGGAGT

18181 CGGATGTTCG AACTTACCGT TCGCCGCAGA AGAGGAAGAT GCCGACGATC CGCGGTCCGC

18241 GAGGTAGGAG GCGACTAAGC GCGTAGTGCG CAACATCGGG TTTAATTTCT CCGCCACTGC

18301 TACGATGGGA GGAGCTAACG GATTTGGGCG TGTCCGCTTT CCTATAGGTC GGAAGACCGA

18361 CCTGAAAAGG TCTATAGCTA ACGACCCGGG CTTTTCAAGT TCAGAGGACG ACGCCGTAGA

18421 ACGACCGTCC ACGTCTCACC GCTCACGCTC GCGCTCCCTC GAAATGCCTG GCGAGAAGCG

18481 AAAACACCAG GCCGCGTGCC TGAACGACTC GGATTCCGAG CTGGAATTAA TGGCGGAACT

18541 GAAGCCGCCC GCCAAGCCGC AGCGCGGCAA GCGCCCTCCG CCGAAGAAGA AGACGACCAG

18601 CACCGCCGAG CTCGCGGATA GCGATGTTCT CGAAGCGGAG CAGGAGCTCA AAAGCGACGC

18661 CGAAGAGTTC CAGTCTACCG GCGGCCCGAT GGCGGGCGCC CTCGCGGAGG ATCCCGTCAC

18721 TTTCAGCGCG CAAAAGGCCA TGGCCTACCT GACGACCGTG TGCGAATCTC TCGATATGCG

18781 CTGGCAGGGA GGCACCATCG AGCCCTTGGA CGCCATTTGG ACCAAGGTCG CCGGTCTCTT

18841 TATGCGCCGA CGGCACCCCG AGTTCCGACT CACCTTCTCC AGCTTCGATT CCTTCTACGG

18901 GCAGTTGGGC AGGTTTTTGG CCGCCATTAT TTACAACTTA GCCGGGTTGG AACCGAAGTT

18961 CGTACCGGGA GGCGCGCACG TTTGGCGACA CGGCTGGAAG GGCGCCACCA TGCCGAAGTG

19021 CTTTCACGGC ATTCCGATGA GCCTTAAGCC TCGCACGGTG GAGTTGAATC CGACCAGCGA

19081 AGCCGGCAAG CGCGCCATCG CCGAGCAGGG CGGTCGCGTG GAGAAGAACC GCTTCGGGCG

19141 GCAGGTGGTG GTGCTCCGCT TCGACAACAA CGCGGTATGC GCGAAAGACA AGGAGCACAA

19201 CGGCTTCCCG TACCCGCACG CGACCGGGAG CTGCGCGATG GTCTTTTCGG ACGCGCAGAA

19261 GGCTCTGAGC GCCATGAAGC ACGACCTGAG TTGGACCATG GCGCTGTACC CGAACGCGGA

19321 TCGCTCGCGC ATCGAGCAGT GCGTGTTGAT CTCGACCAAC TGCAACTGCA ATTACGGTTG

19381 CGAAGCTCCC ATCAGCGGAC GGCAGATATG CCGTATGACC CCCTACAAGC TGAGCGGCAC

19441 CGACGATATC ACCAAGGATA TGCTGGAGAG TCGCGCCGAC ATGAAGGCGC ACCACAAGCA

19501 CCCGCACACG ATGGTGTACA CCTGTTGCAA CCCGCAGGCG CCCGGCGGTT CCAACCCCGC

19561 GGGTTCGAGC CGCGCGCAGA GGCGCACCGA GAAGAGCTGC TCGTGGCGCA TTTCCTACAT

19621 GGATCTCCGC TACGCGTACG TTTTCGCGAA CGAGCTGATC ACCACCGCGC TGGGCACGGA

19681 GGCGGCCACG CAGGTCCGCG AGTTCCGCTG GAACGACAAG TACGCCTACA AAACGGAAGT

19741 CATCGCTCCC GTCTGTCCCG TGTCCCATTC CGATCCATTC GCGTAGTGCC TTACGCGGGC

19801 TTGAGAGGAG CACGCATTCA TGCACGCACG CACGCACGCA ACAAGAGAGA GAGAGACGTT

19861 CGTTCAGAGT GACGAATGTT TATTGCCTTC CTTTCACAAT AAAGGAGTCG CTATGCTTGT

19921 TGTACGTGAT TCTGTGGTAA CGTGTTTATT GCCGTTTCGC GTTTCAGGAG CTCGGCGTGA

19981 CGCTGGAAAT AGGGATTATT CCGCGCGAAC CACGCGTTCA TGCGCTCCTG GTTCCGGTGC

20041 AGGATCTCGC GAAAGGCCGG TTTGTACATA TTCTCGTGGT TGACGCCAAC CACGGTGTCG

20101 ATGATGGGGT TTCCGTTCAT CGGACTCCGT CGGTAGCGAT CGAAAGAGGC GATAAAGAGC

20161 GCGCTGAAAA GCCCGCAGGC GCCCGAGCAC GGGCACTGCA CGGCTTGCGC GGAGCGCACC

20221 AGCTCGATGC AGCGGTCTTG CTGGCTCAGG CCGGTGGCCT TCAGCATCGC GTCGTATTTA

20281 ACTTTGTATA ACTCCAACAG TTTCTTGTCG GACCACCCGA AGGGGTCGAA CATATAACAG

20341 CGCCGGTACT GCGGGTCGTA CGCGAACCCG ATCCAGTGCA TGCCGCCAGA GGACCGGGAG

20401 CCGGTGTTGA CGATAGCCGA CGCGGGGCGT TCCGGGTCCA GAAATCCGGG GAAGGTTCGA

20461 TCGAACACGC CCAGAAAGCG GTGACGAGGA TGCATCGCTG CCACCAGGTC CCGCAACTGA

20521 GACTCCGTGG TCCCCGTCAT GCCGACGCTC TAAGGGTTTA CACGGCGTTG CCTGTGGCGA

20581 AAGGCGTACG GAAGTAAGCC ATAGCTAGCA CGTTACGTTC GGGCTGGTTG ACCCTAACGG

20641 TGTCGAACAC GCCATAGAGC ATGTACACGT AAGTGGGATC ATCCATGGGG TCGAGCTCGA

20701 AGTTGATGAC CATGCTGTGC GAGTTGTTGG TGTACATGGG GTTCTGGCCC AGATCTGTCA

20761 GTTCGCCCAT GTACATAAAG TCGGAACTGA ACGGGATGGT CCACAGGTAG TTGTCGCAGA

20821 GGAACTTCTT GTAGCTGAGG ACCTGTGCGG GTTGGATGGC CTGCTGACCG CAGAGCGGGT

20881 ACGGCCAGTT GGCGGGCCAG CTCTCGCCCT GGTGAGCGCT CCAGACGGGC CAGGAGCGGG

20941 GGGCGATAAA CCCAGAGTTG TTGCGCACGG CTTCCTGACT AGGCTGCTGT CCGCTGTTGT

21001 CCGTGGTAGG GGTGTAAGAC ACGAGGTCGA AGAGGCCGGG CAATGCGAAG TTGGGTCCCT

21061 GGCGCGTCAT GGGGTCGAAA TTTCGCAGGA AGTCGTAGTG GAAGTACTGA CGATCGGGCC

21121 AGAATCGATA GCCGTTATAG ACGAAGTTGT AGTTCGTGGC CATCTGGATC AGGTACCAGT

21181 CCTTGGTGAT GTCGCACTGG CTCATGGTGA AGCCCTCGGT GTCGAGCGCC ACCGAGCGCT

21241 TGATCTCGAA CATGTTAGGC GTGAGCAACC GGTCGTTGCC CGGCCAGTTG ATGGACGTGT

21301 CGAAGAGGAT GCTCATGTTA CGGAAGGTGT GCGAGAGGTA GAAGCCTCCA TCTGAGTAGG

21361 CGATGCTGCC CGAGTACTTG AAGTTGACGT CGTACTGGGC GCCCAGCTGA GGCGTCTCGG

21421 CCGCCTTGAT GCGAGTGAAG GACCACCCGC GCATCCCCTC CCAGGTGCGA GCGGGAATGT

21481 TGATGGTGAG GGCGGTGGAG CCCGCGGGCA CCGAGTATAG AGCGTTTTTG GCTCCCAGGT

21541 AGTCCACGAA GGTCTGATCG TTGGTGGCGT TTCTCAGCAT CAGCTCGAGC TGGTTACTGG

21601 TGTTGTGATC CATGGGCATG AAGTTGGCCA TGAGGTTCAC CTCGTTGTAG ATGATGCTGG

21661 CGCCGTCGGC GCGCAGGTCG TTGCCCAGAC TGGATTGGAG GATCATGTTG GGGTCTTTGC

21721 GCAGCACCCA CTCGTAGGTG TACGAGCCGG AGAGCAGCAG CAGGTTTTTG ATGGCGAAGA

21781 ATTTTTGGGG CACTTGGATG TGGAAGTTGA CGTAGCGACT GTTTCCCAGC AGCTGGGAGC

21841 GGTATTTCAG CCCCCAGTTT CTGTGGTGGT TGAAGGGGTT GACGTTGTCC ATCTGGTCGA

21901 TGGACCAACG CGCACCCACG TTCGTGAACA TGTCGACGAC GTTGGTGAGG GGGACGCGCT

21961 TGTTCATGTA CTCGTAGGTG GTAGGCGCGA CGCTGGTGGC GTCGAAGCCG GAGATGCTAA

22021 ACTTGTAACG GTCGGGCAGA TACTCGGCGA TGTTGGCCAT GATAAAGTTG CGCCTCTGGG

22081 TAGCGCTGAT ATCGATTTCG TAGGAGGGTA CCGTGCCGAA GTAGAAGTTG GTAGTGGCGT

22141 TAGCCACGGC CGTGTTTTTG TCGTTCGCGG CGGTGTTGTT GGTGTAGAGT TTAATTTGAC

22201 TGAGATCGGG GCCGTAGCCT TCTCCCGCGC CTACCGCTTC GGGGTTGAAG GCGTAGCTGG

22261 GCGCGCCTTC TTCGTAACCG TCATTGGAGA AGACTCGCAC CTCGGGGTCG TACTGGTCCA

22321 CGGCCTGGTT CCACAGGGCG AAATAGTGAT GGCGGGACAT CATGTCGGCC AGCATGTACT

22381 GGTAGCTGAG CTCGGTATTC CGGTCGGGCA GCTCGACCAC CACGTTCATG CCCGAACGCT

22441 CCGAGTTGAG GGTGCCCGAG CACACGCCGG AGTCGTGATA CAGCAGGTTA ATGAAGTTAT

22501 CCCTGAACCC GATGTAGTTG GGCCTGAGCG CACGCGTGGT GCCTATGTTA TAATCGTCGT

22561 AGTCCTCGGG AGGCGGCACG ACTATGGTAT CTGGGTACGA GAGGCTGTTG GTGTAGTCCT

22621 CGACGGCCAC CGCTCCCAGG TAATTGGTGC CCGTGTTATC CATGATCCAG TAGGGGGTCT

22681 GCGTGAGGGA CTGGGAACCG TCGGCGGCGA CGGGCTTGAC GTAGGCACCG TAAGCGTAAT

22741 TGTACTGAGA CTTGGCGAAG CGACCGAGCA CGCCGGTGTT GGCGTTTTGT ACCCGTCGCA

22801 GAGGATTTCT TCCGGGTCCC TGGTTGGGAT TGGGGAAGAC GCCGGAAATC TTCGTCACCT

22861 GCGCCGCCGT CGTGTCTTTG GAGGTGCTCG TGTTGGTATA GACGTTGGAC AGCTGACCGG

22921 AGGCGGACAC GTTCTGCCCG GGTGCCGTCT CCGACCAGTT GTTAAACATG GACTCCTTGG

22981 GAGCCAGCGG GTTGTAAGCC GTGCCGCAGT AGGGCTTGAA GGACGGCCCT CGGTCTAGGA

23041 TTCCCTTGAT GTCGAAATAG GTCGACCCCA TGTCCAGGAC CCAACCGTCG CCCACATTGA

23101 TGTTGTACCG CACGCGGTAG CCCGTCGACG TGTCGTCGGT TTGGATGGGG TAAAAGCGGA

23161 TTTGCAGCCG TTGAGCCTTT TCTGTCGTGA CATTTCGGGT GGGCGCCACG ACCGTCTGTC

23221 TGAACTTGTT TTTCAAGTCA AAGTAGCTTC CGGTGGCGGA AATGAACTGT TGGAGGTCCT

23281 CAGAGAGGTA TTCGCGCGTC CCGGGGCCCG CGATGTGAAA ATACTGGAGC CGCGGAGTCG

23341 CGGTAGTCAG GTCGGGCGTG AGGGCCGCCA TGGCGGTGCG AACCTAGACG AAACGCGACA

23401 TACATACACA ACCCGTTTAG TAACACAATC GCCTACTGCT GACATTCACT CCGGTGCCCG

23461 ACAAGGTGTT GAGCCTCGCT CTCCAGGAGC CCGCTCTGGC TCGCTTTCGC CTTCGCTTCG

23521 CGGGCGGCGG CATCTCTAGG GTAGTCGGGA CGTCCACCAC GGGCACGGCG GGAGCGGAGG

23581 ACGTGATGGG AGGGTTCACC TCCGTCACCA TCTCGGGAAT CGGGCGTGTG GTAGGCACGG

23641 GCGGCGCCGG TACCGTCTGC GGGACGTGCG CAGAACCCTG CGGCGAGACC TCGGCTCCGG

23701 CGGCGAGTTG GGCCTGGAGA GCCTGGATCA GGCTTTGGAG CTCCGCTTGC GTGGCGGGAC

23761 CTTCCTCCCC GAGAGCCTTA CGGCGAAGCT TCTCTAGATC TTGTTGCAGC TTCAGGCGCC

23821 CGATGTCCGT CAGGGCGCCG AGCGTCTCAC CGGCCAGCGA CGCCACGTTG CGCACTATGC

23881 CGCTATCCTT AAGGCCCTGC TTTATCTGGT TAAAAGCATT GGAGTGGACG AAGCGGTTGC

23941 CGGTGTTGTA CAGCCATCGT CCGGTGGAGC TCAGCGCGCT CGAGAGCCGC GAGCCCACGT

24001 TGGACCAGTT TATGGCACCC CCGCGCAGGG TGGAGGTTCC CAGGTGATGT TCTCTCAGGG

24061 CCCAGGACCC GACGTGAGGC GACAAGGCGG CGTAGTCCAT AACGACGGCG GCGAGTGATC

24121 GATCGTCTAC AAAATTTTTA TTGGAAACGT CTTAGACGAC AGTCACACAG TCAGCACTTC

24181 GGTTCTCTCT CTCTCTCTCT CACTTGTTGC CATACAACTT ATTGAATTGT CTCTGCTGTT

24241 CCTTAAGGCT CGCGATGCCC ACGGCGGTGC CGGCGATGCC GGGGATGGCG CCGATGGCGG

24301 CGGCGATCAG AGGAATCAGA GCCGGGAAAA AGCCACCGCG CATCGATCGC CTCGCCCCGG

24361 TGCGACGCCG CCTCCCGGTG CCGGTGAGCG GGACCGCCTC GGCCGCGGTG CCCGTAGTCA

24421 GGTCTCGCAC GTAGGCCGCG GCGCCGCTGG CGGCGGCCGC CTTGGCAGCT TCGCTCGCGG

24481 CGACCGAGGG GTCCGCCGAG CGCCAGGCCG TGTAGTTGCC CCTCTGAAGG CGTTGCGCCA

24541 GATTTTGCAA GGCAGCTCTC TCCGAGGCGC TGGCCGTGCC GCTCACGGGC ACCGAAATGG

24601 ACGAACGCTT CCGAACGCCG CTGCGCTTGC CGCTGCGCGA GCGAATCTTG GGCACGGACA

24661 CCGCTTTCTT GCGACGCTGC TTGGTGCTGA ATTTACGCTT GGAGGCGGCG CGACCCCCGG

24721 TCAAAAGCAC GGCGGGCATG GTCGAAGAAT TAGCGACGCG TCGGGGGACG ACGAAGGCGG

24781 CGCTGGTCCG TCCTGTCTGT ACAGGGACGG TACAAAGTCG TTACCAAAGT CACGAAGGCT

24841 ATAACAAGTC GTTCGAACTA GAATCGGTAG ATGGAGGCGA CGGTAGGCGA CGTAGTCTGC

24901 GCTGCGACGA TCAACGACGT CTTCGCATGC TGCTGGTGCG GGTGCGCACG ACGACCAGCC

24961 GGCTGGTGGT CCTCGGCCGG GAGCGGCGGG AGCCGCGTCT CCTCCGGGTG CCCAGACCCA

25021 GGAGCGAGCG CAGCGTCAGA GGGCGAGCCC TGCGACGCAT CCCCACCCCG CGCATGGATG

25081 ATGATCTAGA GCGGCGACGC ATACCCCATC CTGTGTTGTT ATTCGGGGAT ATCAGAATGG

25141 ACATGTTCAG TCCTACTGCA AGGTCGCGGA ACTCAGAACG GTCGGCTGAA CCGTCGCGAT

25201 AGACTTATAC ACGTAGGGTA TCGGACGACG CTGGTCGTCT GTGATCAGCA CGCGCTGCAG

25261 TCCGGGGAGC GAGGTCTTCA CAGGCAACAC ACCCTGCTGA ACGACGGCGG GTTGGTTATC

25321 GCACACGGAC GAAACATTCA TGGGGGGCGC TTGCTTCAGA ATCTCGTTTT CGGGAAAGCG

25381 GTTGAAGGCG GCTGTGGCCG ACTGGCAGGA ATTTTCCAGG CGCTGCACGT ACGTGGACGC

25441 CGCCTGGTAA TAAACTTTAT TGTAGGTGGG GAACAGGTTC ATGCCGACGA CCGGGCAAAG

25501 GTTGGTCGTG TTATCTTCCT TGAACCCGGT AGGCGCGATA AAGGTATCGG GCAGGGACGT

25561 GTACATCGCC CCGATCCCGC CCGCCATATC GGGCACCGTC AGCAAGGTCG TCTGATTGGC

25621 CGGCGAGTTA GGCACGTTGT AAGCCAACAT CCACGATCGA TACACCGTCA CGGGTTTACC

25681 CGTCACCTGG TCGTAAATGA CGTTATACGA CACGCCCGCG CTGTCATGTA AAAGGGGAGC

25741 AGCGTTGTCG AGCTCGATCA CTTCACCGTC AGCGTCGTTC ACGTCGACGG AGTCGAGGTC

25801 CAGCAGAGCC GGGATATCCC CTCCCTGCAG ATCCTCGTAG GTAATAACGA ACCCCTTCGA

25861 GTAGGGCTCG CGCTTCCCTA TGCCCAGGAG CAGGCTCAGG CGGCTGTACG TAAAGTCGAT

25921 CGCGCATCCG GGCAGCAGCA CGATGTCGGG GTGGTAACCC TTGTACACGT ACGTGCCCGG

25981 AGTTACCAGT CCCGTCACGG GGTCGCGGAG CAAGCCGAAG TTGCGCGTGT CGAACTTGAC

26041 CCCGATGTCC GATTTTTGCA CGTTGTTCTG GCGCCCCTCG CTCAGGTAGA GCTGCACGAT

26101 GCCCTCGTTG AGCAGGTCTA TCAGTTCGCA CAGCGCGTAG TTACCCTCGG GTATCGTCAG

26161 GTCGTACCAC TTGTACTGCG CGCCGGGCAC CGAATAGCCG CTGCCTACCG CGCTCGGAGG

26221 AGCCGTGCTA GTCGGCGGGT CGCGCTTCCA CATCATGCGC ACGCGCACGC TGTTACTCTG

26281 GAAAAAACTG CTCACGTTCG GGCAGTTGGT GCGCACGGCT GTTTTTAGGT CGCCGCCCCA

26341 GCAGGAGCGG TTGTCCAACT GGATGGACTC GGTGGCGGCC GTGTCCGCGT CCAGATCCTG

26401 GTTATGGATC ACCGTGGTGC GGAAATTGCT ATGGTTGGCG TCTTTGTTAT ACGTATCGAT

26461 ATCGCTAGCC TTGTTGTCTA CGTAAAACAG CTTGGTGGTG TTACGACACG GCGTGTAATC

26521 GCGATACTTA ATGCTGTTCC GTCCCCCCGT AGGGGCCATC ACCCGCTGAA GGGGCATATA

26581 CAGCTCGCTC TGACCGTCGC TAGAGGGACA GCTCTGCGCG GACGCGGCCG GAGGCGGATA

26641 GCCGTTCACC ATCGCCGGAT AGGTCGAGGG CGTTAACTCG GTCGGCGGAG GAGGCGGCGG

26701 AATCGACGTC GGCGGCTGCA ACCCCCACAT TGCACCACTC CTTGTCGGAG ATCCTGTCGA

26761 AATGGCGGTA AGTGTCCCTT AATAATGGTA TCTAGCTCTC GTTAGTAAAA GCGTAGGCGC

26821 TTGCCTTGAC GGAGACCCCT CGCTGCTAAC GCTCGTCTAG CCAGTCCCGA CCCTCTGATT

26881 CCGCGAGCGG GCCCGTAGCC CGCGGACGCG TAGCCGGCGC TAGCCGGCCA TGGGCAAGGC

26941 GGTTGTCCCG TGCCCGCAAG TCCCTTGGCC AGACCTGTCG AACGGCGTGT GCCCACGCTC

27001 GGCTTCAGGC TGTCGAAGAA GCCGGCGCCC CCGGTGCCGC TCAGCTTCAG CTGCCCTAAG

27061 GCGTCCGTCG CCGAGCTCAC CGAGGCGTTG CGACTGCGCA TCGGCGCGTC GTCGGGCAGG

27121 GGGGTGTTGT AGGGAGTGCG CTCGGACACG TTCAGCGCGC CGTCGAAGGC CTGCGGGAGG

27181 CGGTGATCTG CAATCCAACC GGCCTCCGAC TGCACGCCCA AAGTCGAGTC GAAAAAGTTG

27241 ATCAGCGAAT CGTAGTCGGC CTGCTCTTTC TCCGTAAACT TTTTCTGGGG CGGTGGACGG

27301 AACGGACTGT CGTCAAAGTA CTCGTCCCGC GAGGGACCGC CGTACTCGAG CTCGCTCGCA

27361 AAGTCACTAA AATTGGGCAT CATTTCCGGG ATCACAAAGT TGTCCGTATA AAGGCCCGCT

27421 GGCGGCACCC AGTGAGGATC CAGCACGATC GTCTTGTAAT AGTAGGGATT GCGCGCGCTC

27481 GCCATCAGCA AGTAGTTCTG GAGCTTGTCG ATGAAGACCT TATTGCTCGT ATACAAACGC

27541 GGATCCACGT ACTGCGCCAT CGTGCTGATG GCTATGTCCG AGTTGACGCC GTCTTTCAAC

27601 GCCCGTCTCA GCTGACGCAT CAAATACTTA AAAAGACTCT CCTGCTCGGG GCTCATCTCT

27661 AGCGGCCCCG TCTGCGGGAT GGACGGCTTA TTCTGCAGCA GATAGTTGAG CGTGTCCTTG

27721 TACTGGTTAA ACTTGAGATT CAGATTGGCG CCCGCGTCGC CGAGTTCCAT GATGGCGTCG

27781 CCCTCCGCGT CCGTGTCGGC CCTAATGGCC TCCTTGTACA GACGCATCAG GTTATCGAGG

27841 TAGCTACCCG GCTCCATCGA GCCGTAGTCG TAGAAGGTGC TCACGAAGAA GAGCAGGGCG

27901 CGCGTGTTAG GCGTCAGCAG CGCCCCTATG CGCGGATTGT TTACGGCATC CCACCGCGCC

27961 CCCCAGATGT CCTTCAGGTT GTCGAAGGCG TTCGTCAGAT TGATGTTCTG ACTCTGACCC

28021 CCCATGTTAA TCTGCAAACT GAAGGAGCCG CCCGAGCTGT ACACTTCCGC GTTCGGAACG

28081 TTATTCACGA ACAAGCGGAG CAACTTCTTG AAGCCCTCAA AGTTGCGTTG CCCCCGGTCG

28141 ACCGTCTTAT ACAGACTGTC GAAGAAACGA GTCAACATGG AAGCGTTCGC GATCTCGCGC

28201 GTGCGTGCCA GTTCGTCCTG CAACACTTTA TTTTGACCGT GGATGACGTC GTTCACCAGA

28261 GCTTGAATGC TCTCCTGAAC GCCCATAGAG TTCCACGTGT ACACGCGGTT CAGCAAGTCC

28321 GAATGGATCG CTCCCATCTG ATCGGGGTAG ATGGCGCCCT GCTCTGCCAA GCCCTTGACC

28381 ACCTCCGCTA CCCGTTCCGA AGTCCCGTCC ACTTTAGGGG GCACCATCGC CGTTTGCAAT

28441 TTGATCAGGC GGTTAGCGTA CGGTTCGTAA CGGAGACTGC GGGCATCCTT GCTGTTGGCG

28501 TGGGAGCTCA GCGCGTCGGC CACCTCAGTA CGCCCCACCG GCGCCAGCGC GCCGAAGACT

28561 TCAGTCGAAC TCATCCTCCT CTCCGCTGTC GGAAAACTCT CCGTCGGCCG GAAACTCCCC

28621 TTGGGGAGGT CGGTTCTCGA GCGCCTGGGT CAAACCGAAC ATGTACTCGG CGTCAGTCAT

28681 CTGCGGAATC TCGCTCATCC GCTTCTGACG GAAGGTTACC GGCCTGTTGT TGTACACGCC

28741 TAACTGCACC CCCAAGTCCA GCACCGCCTG CATCACCCGG TAGAAAAATT CCTGGGCCTT

28801 GGCCATCTGA GCCATGGACG GGTAGCCGTT CTTCGCGGCC AACTTGGCGT ACTTCATGCT

28861 CAGCTCGACC ACCGTCGTGC ACACCGCCGA TACCTGCTGC TCTATGCTCA GGTACGGGTC

28921 GGTCACGATC ATGTACGCCA TCTCTATCAG GTCTCTCAGC CACGCGTTTT TCTCTTCCGC

28981 GATCGCCCAT AGGCGTTGCG CGAGCGACGT ATTTCCCGCG TGCTGCGCCA CCAGCACCAG

29041 CTGCGGGTTC AGTTTGACGC GCCCGTCCGG GTGCTCCATG TACGTCTGCA CAAAATCGTA

29101 CAGGTAGTAG ATGCCCGCGC CGACCTCCGG TCGGGAGAGC GCCGTGCGGA TACGCGTCTG

29161 GTGACACACG TTGCGCATCT CCTCCCCGAA AGCCGTTTGC TCCGAGGCGC GCTTCAACTC

29221 GGCGGCCTTC ATGTGATTGA CCGCGGGCGA GAACGTCGGG TCACCCGCGT CCGCCGCGAA

29281 GTCTTCGGGG CGCAGCACCC GGTTCCTGTC CAACTGCACT ACCTGTCCCG AGCGATACAT

29341 CATGTCCCGA TCGTAATCGC CTTGAGGAAC CGTGTCCGGC TTCGCCTTAA ACAGGTTCAC

29401 CTCGGGAATG GCCCCCTTTC TAGCCGCGTC ACGTTCGTAC ATGCGGGTCG GGTCTAGCGT

29461 GGCTCCCACC GCCATCCCAC ACGACGGAGG CTCCTCTGGC ACGGCCCCCG CCGCCATCTC

29521 CGGAGGCCCC GTCGCCACCG GAAGCGCGTG CTGTGCCGGG TATCGGGGAG GGTCGGGGGG

29581 AGCTCGCGCG CCGCCACCCC CGCCCGCGCT GCGGTCCTGG TACGGCTGCT GGTGCTGGTA

29641 GGCTTGACGC TGCTGATCGT AGACGGGCGG CAACTCCTGC TGCTGCTGGT GCTGCTGCGA

29701 CTGACTCGAT CTTCCGCCGG AACCCAAACT CGCGTTTCGC ACGTTTTGAA GAACCGGGTG

29761 CATTATGCAG CCACGCGCGT GTCTAAAAAC GTCAGGGGTC GATTTCAGGC TTACAGGATC

29821 TTCGCGGAGC TCACCGGCCA AAGCCTAAAC ACCGTACGCT TCTGCGAACC CGTCATCGCC

29881 GCCGATTTCC CGATGGTGCG CACGCAACCA GCCCTCGTGC ATCCCCGTAG CGTCTTTCTC

29941 GTCAGCCGAA TCTACGACTA CAGGCTCATG CAGCTGCGCG ACCTAACGCC GGGGGGAGCC

30001 AACGTCGCGC AGAACCCCTA CAACGGACTG CCGCCCCCGC ATCTGTTGCT CGGCTACCAG

30061 TACATGCACA GGACGCTCAA CAACTACTTC TTTGACAACC GCGTCTTCAT GCAGCTCGGA

30121 TTCGAGAGCC CTCCGACACA ACGACCGCGC AGGCTCTTCT GGACCTGCCT CACCGACTGC

30181 TCGTACTCTG TCAGCGTCGG ACAGTACATG CGCTTCCTCG ACCTAGACAA CTTTCACGGC

30241 ACCTTCACCC AGATGCACAA CGCGGTGCTC ATGGATCGCA TCGCCGCCGA CATGCAGGGA

30301 GCGTATCTCC GAGGCAGAGG AGTCGTCGTG GGTCGAGACG GACGGGTCAT TCCGCAACCT

30361 TTCAACGCCG ACGATCACAG CTATCTGACG GGTAGCGGCG CGAGCGGGCT CAGGGACGAC

30421 GTCGTCCTTC GCACGGCTTC GCATCGCGAC GCCGCGATCC TCGCGGCCAT CCGTTACCTC

30481 CGCGTTGCGC TGTGTCACTA CCTATTTTGC AACGCCTACG ACCTCTTTAC CACCGAAAGC

30541 ACATATAGAT TCTTACCTGG ATCGGAAGTG TTCGCTGAAG ACGATTGGCT CAACCTCTTC

30601 GTCGAAGCCT TTGGCGAGCT AGACACTCAG CGGCTCGTCC GCGCGGTCGA AAACGATCCT

30661 CAAGCGGGAT GGCTCGGGCA GGATCCTGCG TCCATAATGG CGAGATGTCT CGTTAGTACT

30721 CTGGCTAGCG ACACCTCGCT CTCGGGAGGC GCCATTACCT TGCGAAACCG CAGAGTCACC

30781 GACAGATCCG GATTACGGCC TCGCGACCGT CACGGACGCG CCATCACCGC GTCCCAGATC

30841 CGCCAGATCC GACGGAGGGC GGTAGAGCGC TTCGTCGACA GGTTGCCGAG GCTCACGCGT

30901 CGCCGACGGC GCCCTAGACC CCCCTCCCCC CAACCCCCCG AGGAATACCT CCCGGAACTG

30961 GAGCCCTTCC CGCCCGAAGA AGAGGAAGAG GAAGAACAGC TCCTCGACGA GGTCGTCCGG

31021 ACCGCGCTCG AGGCCATCGA CGCCTTACAG CAAGAGCTCA GTCGCACCGC GCAGAGGCAC

31081 GACCTGTTCC AGTTCGCCAC CGCCTTCTAC CGTCTGCTCC TGCAGACACA GCAATCCGAC

31141 GTGGCTCTCG TCACCGACTC CTTCCTCAGG AAGTGGGTCC TCTACTTCTT CCTCGCGGAG

31201 CACATCGCCT CCACGCTATA CTACCTGTAC AGTCACTTTA TCAACTACCG CGAGTTCAGG

31261 CGCTACGTGG AAATCGACAC GCTGCAAGTG CTCATCGTGG GTTGGGATGT CAACGCGCAG

31321 CAGGTTTTTA AACGAATTTG GAGCGAGCAG TCCAACTCGT CGCGCATTTT CGAAACCCTA

31381 TGGAACCGCA TCCTCCGCGA CTTCCTGCTA ATGGTCGAGC GCACGGGACA ATTCGAGGGC

31441 ATGGACGAAA CTGATCAACA ACTCTTTCTC TCCGATATCC AATACAGAGA CAAGTCGGGA

31501 GACATCGACG AGGTCCTCAA GCAACTCAAC CTTAGCGAGG AGCTCATCGA GAGCATCGAC

31561 ATCAGCTTCC GACTCAAGTA CCGGGGCATC GTAGCCATCT CCACCAATCA GCGCATTACC

31621 GACAACTTAC GCCAAGTCCT ACAAAACAGG AGGGAAGAGC GCGACTTCCA ACAGCGATAC

31681 CATCCCCAAC AGCGCCGCTA GCGTCATGGA ACACGTCGCA GCGGGACGTC TGCGACGCTC

31741 CATCCTGGGC GAGCACTCTC ACCTCGTGCG AACGGTACCC AGGCGTCAGC GGAACGATCC

31801 CGACAGCGCA CCCGGGCGCC CCCCATCGAC CCTGTTCAAA ACCGTACCCG GAGACGTGTT

31861 AAAAAATGGC ATTTTCTACA TCGAAGGAGA GCCGTTCAAA TTACAATGCG TGCCCTTCGC

31921 GCGGGGCATG AAAAAATTCC TCAAGCTGCA CAAGTTCCTC CTTACCGAAC GCAGTCGCCA

31981 GTACGACATT ATCGACTACG CCTTCTACGA GCGTGACCCG ACGAACTCCC TGCGCGTCTT

32041 CCGGCCGAAG TACATCGGCG TCCTCAAGTT CCTCGGAAGA AACGCCGACA TCGAACGCCT

32101 CTTCGAGGAC GAAGCACCGC TCCTACCTCC CATCCTGCTA GCTCGGGACG CCCGTCGAGA

32161 GCCCGAACGC TGGCTCTGGG TCCTCTCGCG AACCGCCGTC CAACACTGTC CCACCTGCGG

32221 GCGACACTGG GTGCGAAACC ACGCGTGCAA CGAAAGGCGA TCCGCCTTCT ACTACCACGC

32281 CGTACAGAAA ACAGGTAGCG AAATGTGGCA ACACGTCCAT TTCTCGTGCC CGGCGCAAAG

32341 CCCCCACTGC AAACAGCTCT TTCTCACTTA CGACATCGAA ACGTACACCG TCTTCGAACA

32401 AAAGGGGAAG CGCATGCAAC CGTTCATGCT CTGTTTCATG CTCAGCGGAG ACCCCGCGCT

32461 CGTCGAGGTC GCGCGCAAAA TAGCGCTCGA AGACACCGAC GTCCGACAGC TCGACGAAGG

32521 CTTCTACTGG ATCGACCCGA AGCCGGGCGA GGTCGCGCGA CGATTCCGGA CTTACCGCAC

32581 ACGACTCCAA CAGCACTTCG CCGAACACCT CGTGCGCCGC TACTGTCGAG CCAATCGAGA

32641 GTTCTGCGGA GAACTCATGA GCGACGGAAA CTACACATCC ATCTATCACA TCCCGTACGA

32701 AAAATTCCTA CAGCCCAGCA AGCCTCTCAC CTTACCGTCC GATTTCTACT CCGTAGACGT

32761 CATCGTGTTG GGTCACAACA TCACCAAGTT CGACGAACTG CTCCTCGCCA CCGAACTGGT

32821 CGAGCGACGA GACCTCTTCC CCGACGCCTG CCGCTGCGAT CGATCCTTCA TGCCTCGCGT

32881 CGGCAGACTA TTGTTCAACG ACATCCTATT CCACATGCCC AACCCCAACT TCAGCAAAAA

32941 GGACCCCACC CGGTTACACC GGTGGGTGAA AGGCGTCGTA GACGAACGCG ACATGCGGTC

33001 CGTCTTTGTG CGTTTCATGG TGCGCGATAC ACTCCAGCTT ACCAGCGGAG CCAAACTCGC

33061 CAAAGCGGCG AGCGCTTACG CCTTAGAACT GTCCAAAGGA CACTGTCCGT ACGAAGCCAT

33121 CAACGAACAC GTGTCGCGCG GACACTACGA TCGGGACGCC GACGGCTTCC CCGTCGCTCG

33181 TTACTGGGAG GACGCCTCTG TCCTCGATGA ACAAAAACAA CTGTGGAACC AAAATCACCC

33241 AGGACAGCCG TACGACCTCG TACGAGCCTG TCTCGAGTAC TGCATGCAGG ACGTGCGCGT

33301 CACCCAAAAG CTGGCGCACA CGCTCTTTGA AAGCTACGAC CGATACTTTA AGCAAGAGCT

33361 GGGCATGCAC GGCAATTACA ACATCTTCGT CAGGCCCACC ATCCCCAGCA ATACACATGC

33421 CTTCTGGAAG CAACTTACCT TCTCGCAGTA CGTTCAGGAA CAGCTCGACA AAAGACAATC

33481 CAAACCCGCG AAACGAAACA AAAAAGGCAA CAAAACCAAC AAATCCATAC CCACCGATTA

33541 CGTGGCCGAG GTTTACGCTC CCCATCGACC CATGTTTAAG TACATTCGCC AGGCGCTGCG

33601 CGGGGGTCGA TGCTATCCCA GCGTGCTAGG GCCGTTCACG CAACCCGTAT ACGTGTTCGA

33661 CATCTGCGGT ATGTACGCCT CCGCCCTCAC CCACCCCATG CCCCACGGTA TGCCTCTCGA

33721 TCCCAAGTTC ACGGCGGCAC ACGTGGACGA GCTGAACGCT ATATTGCTCC AGCCCGCACC

33781 CATCAGCTAC TTCGACGCAC GCATCAAACC TTCCATCCTC AAAATAGAAG CCTACCCGCC

33841 GCCCCCCGAA CAGCTCGATA CCCTCCCTCC GCTGTGCAAC CGCAGAGGCG GTCGCCTCGT

33901 ATGGGCCAAC GAGGTGCTCT ACGACGAGGT CGTCACCGTC CTAGACATCA TCACGCTTCA

33961 CAACCGCGGG TGGAAAGTCA CCGCCCTGCA TGACGACATG AACATTGTCT TCCCCGAGTG

34021 GAAGACCATT TGTGCGGATT ACGTCAGCAA GAACATCGCG GCCAAGGAAA AAGCCGATCA

34081 AGAGAAAAAT GAGGTCATGA GATCCATCTC GAAAATGCTC AGCAACGCCC TCTACGGGGC

34141 CTTCGCGACC AACATGGATA CCACGCGTAT CGTGTTCGAA CAAGACCTCA CGGACAAGGA

34201 CAAAAAGGAG ATCTACGAGG GCACCCAAGT GGTCAAACAT GTCACGCTTC TCAACGACAG

34261 GTCGTTTTCG GGCAAAACTC TTTACGAGAC GGGAGATCCA TTCTCTGCGC CATCCTTATT

34321 AGCACATTTT AAACCCCCAG AAGAGAGCGA TGACGAGGAG GAGGAGGACA CCGAACACTG

34381 CGAGAGTACC AGTAAGGACG AAAACGTCGT ACTTACCGCC GAGGAGGCAG ACCTCTCCGA

34441 AGTCGATCAG GAGCTGGAAG AAGCGCTCAC CTGCGGCCTT TATATAGATG ACGGGCGACC

34501 GCCGGCAGAA ACAAACCCCG CCCACTCGCG AGCCACCGAG ACCGCATTCA AGCCCATTCG

34561 ATTCCTCGAC GCGCCACCGG AGGCGCTCAC CGTACTCCAC CTAGAAAGCC TGGACAAACA

34621 AGTCGAAAAC AAACGTTATG CCACCCAAAT AGCATGCTTT GTTTTGGGCT GGTCACGAGC

34681 ATTTTTTAGC GAATGGTGTG AAATTTTACA CGGACCGGAC AGGGGCACAC ACATTCTCCA

34741 CCGAGAGCTG CAAACGCTCT ATGGGGATAC CGATAGCTTA TTCCTCAGCG AGACGGGCTA

34801 CGAACGCATG AAGACACGAG GAGCTCACCG CATCAAGTCT AAGAGCACAC GCCTCACGTT

34861 TGACCCCGAG AAACCAGACC TCTACTGGGC ATGTGATTGT GACATCAAGT GCAAACAGTG

34921 TGGTAGCGAC ACCTACAGTT CCGAAGCGAT TTTCCTCGCC CCAAAACTCT ACGGTCTCAA

34981 AGACGCGGTG TGCACGAACC CTCAATGCGG CTATGTGGGC ACCGGGAAAA TTCGATCCAA

35041 GGGTCACAAA CAGGCGGAGC TTATTTACGA TACCCTCCTA CGTTGCTGGA TGCGCTATGA

35101 AGACCAACTG TTCGGAGCAG ACAGCCGCAT TCCCGAACTA CACACCAGGC GAACCATCTT

35161 CAAGACCACG CTGCTTAACA AGGTCAGCCG CTACGAGCCA TTCACGATCC ACAACGAGCA

35221 GCTAACGCGG ATCCTGAGGC CCTGGAAGGA CCCCACGCAA TATCAGTACG GTAACGCCCT

35281 CTACCCCTAC GACACCGAAC ACCCCAACCC GCGCACGGTT GAGGAAGTAC GCCACGTGTC

35341 CGTACCGGGC GACGAGGAAC CGCTTGCGCC GCTGCGAATA GACCCCTATG CCTTCCTAAC

35401 GGCAGAAGAG TGTGACGAAA TACTCGAGCT CCTCGGAGAG ACGGATGAGC GCGATCCCTA

35461 AGAAAAGAAA AGCCTGGCAG TATCGAGAAG AAGACTACCT CTCCGGCGAA CAGTTCTACA

35521 ACCGAGTCAC GGGATGGTAC GCAGGAGCCA CCGATCTAGC GCCCCAGCTC TTCAAGGAGC

35581 ACCGGTTTCT CCCGTTCGAC GAGTTCTACA GTCTCGGCGG AACAGACGCC AAGTTCCACG

35641 AACTACAACA AAACGTCGAA CAACAAGAAC ACCACGACAG GCAGTATCTC AGAAACGGTC

35701 AACTACAGTC GCTCAACATG GGACGTCAAC CCGTCATCGG CGTCATCTAC GGTCCAACGG

35761 GATCCGGAAA AAGTCATCTG CTGCGCGCAC TCATCTCGTG CAACATGCTG CAACCTATCC

35821 CGGAAACGGT TATCTTTATC ACGCCGGAGA AGAATATGAT CCCCCCTATC GAGCAAACTG

35881 CTTGGAACCT ACAGCTCCTG GAATCCAACT ACGACTGCAG GGACGACGGG ACATTCGCCC

35941 CCAGGACTTG CACGTTCAGA CCGGATTTTA TCGAGATGAC CTACGAAGAA GCTACTACAC

36001 CGGAGAACCT CAATATCGAG AACCCCAACA ATATCTATGT GACGGCTTCG AAGAAGGGCC

36061 CTCTAGCTAT CGTGATGGAC GAATGCATGG ACAAACTGTG TTCGGGTTCT AGCGTCTCGG

36121 TGCTCTTTCA CGCGCTCCCC TCCAAGCTTT TCGCTCGCTC TGCTGCTTGC TCCGCTTTCT

36181 ACATCTTTGT TGTTCTCCAC AACATGGCTC CCAGAACGGC CATCGGTAAC GTCCCCACTC

36241 TCAAAGTCAA TGCCAAAATT CATATCATGT CCTGTCACAT CCCCCAATTT CAGTTCTCGC

36301 GCTTCCTCTA TTCGTTCGCG CACAACATTT CGAAGGACAT CATGGTCCTC CTCAAAGCGT

36361 ACTTTGCCTA TCTCCAGCAG AACCAAAAGT TCAGCTGGAT CGTATACACC CCAGACCCGG

36421 TCTCCGACTC CTTTCGCTGG TGCACTCTAG ACAGAAACTA TGATATCATA CCCCTCAATA

36481 TAAACATTCA GGAAAATTTC CTTAAAGCCG CTAAACTAAT CATGCGATTT ACAGATAATC

36541 ACAAACCCCA TTGGGAAAAG CGTCGTAAAC TAACGGTTCT CGAAACCATT TCTCCCTCAG

36601 ACTCGCCAGA CGAGACAAAA AAGGCCTAGC AATCACGAAC AGATGTCAGA GCTCGCCTTC

36661 CATCTGCAGC TCAGGTCCAT TTTCACTCCC TCACAGTGGG AAGAAATCGA CGCCGAGCAG

36721 TATAAAAGGC ATGCCAACAA CGCCGCCGAA CTGCTCCTCG AAGCCAAGCG CATCTATTGC

36781 AATTACGGTC TTTATCAAAC CCTAGAGTTG CGCTCGAAAC ATGGTCACTC GCCGCTTTCC

36841 AACTCGAGGC AGCTGGCGTC CATCGCTGCC AAAGAAGGAA TGCATCTCAC CTCTCTGGTG

36901 AACGCCATCA CCTCCTGGCT AGACGCATTC CCCTACAACC CCAGGAAAGA CTACGTTAAC

36961 ACTATGTACG TAGTCGGAGA CGCGTCCAGT TGCGCTGATC CATTCGCCTT CTCCCTCGTA

37021 CGAGCTTTCG AGTGTGTGCT CATGGGCGAC ATGAATCAAT TTGACATGAA AGAATACGCC

37081 AGGGTGCAGC GCGAAACCAA GTTCCTCTAC TTTCCTCTAG CTTTCCACTC CCTTCCTTTC

37141 CAAAGCCCGA CCGTCAACAA CATGCTCCAG GGACGAGAGA CGACCATCGC AAGCGACGGT

37201 GAATTAGTCA CCATCAAGCC GACCAAATGC TTAGTTAGAC TCAGATCCCT CCCACACCCC

37261 GATCGACTGC CCACCAATAA AAACCAGCAC GTCATCATCA ACTTCGAAGC TCCCACCGTA

37321 GGTCTCGCAT TCGAGGCTGC TGAACTCGTC GGGTACATAC GGAGACTCAA AACTCACGCC

37381 GCGACCTCCA ACGACGTCCT CGAATGCTGC AACCCGTACG GATACCTATG TTCCAAGTCA

37441 TCCGCTGGTG ATATGTGCTC TACATGTTCC GAATATCACG CTGATTTTCT CTCCCTTTCA

37501 GACGACTACT AAGCGTGCGA AACGAGAAGA GGATTCGCAC GAAGATCAAT TCTTCCTATT

37561 CGAAGACTCT TCGCAAGAAG CCCCTCCCAT GCATCCCCTC ACCGTTCTCA TCGAACAGCT

37621 CTTCGAAGAG GGCATCGCGC ACGATCTCCA ATGGACCTTC CCCACCAAGC ATCTGCTGCC

37681 TGCCCACGAG CGAGAATACG TCCTGTCGGT CCTCAGGGAG CGCTTCGGAC CGTCACAGAG

37741 CCTGTTCCTC CAACTGCCTC CCGAAGCGTC CGACCCTATT TCCAGCGCCT TCTACAACCC

37801 CAGGGAAAAC TGGTTCTACC AACTCCTCGA AAAAGAAGGC TACAACGCCA GAGTAGCATC

37861 CGCGACCATC GGTGCATGGC TCAAGGGTGA ACTCAACACG CTTGTCCTGT GCGGTGACCG

37921 ACACTCCAAC GCCAAAATGC TCTTCAACGT CATTTCGAGC TGCTTCCCCA TGGCTATCAC

37981 CGACAGTGCG ATCAACTCGC TCGACACACT CGCAGAAATC TCCCCCATCA CGCCGCTCTA

38041 CTGCCTGCCC TTCGTGCAAG AGAGACCCAA CGCCATAATG CTACACTTCA TGGAAGGAAA

38101 CTTTCTGAAC ACCGTCATCA AAGGACAAAT GAGGCATATC CCTCACACCT CTGTTCTGAT

38161 ACACTGCTCG GATCTCAGTA TCGCGGACAG CTTCTCCAGC CGAAACACCG CCATCCTCTA

38221 TCTCGTACAG GAAAACAGAT CGGTGCCCGC CTGTCACTCT CCACGAAAAG AACTAAGGGA

38281 CTTGGTTTTG AACGCGACCG GGTTACCATG CTTACTCAGT ATCCATTGTA AGAAAGACCA

38341 TGAGATTTGT GATAATTGTA TACGGGCTTC GCCCTCTGAT GCACTCTAAT AAATCTTGTT

38401 TCAGATCAAC CCCGTGTCCG ATATGGTGCT TTTGACCGGT TCGAGGGCTT CCCCTCCTCA

38461 CCACCGCCAC GACTACACCA TCCTCTGTCG ACCCGCCCTA GACATATGCG ACGCCATTCA

38521 CTTTTTCGAC CTACGGTTCC TCGAATTCAT CAACGGGCGA GCCCTCCTAC CCTTCCCCTC

38581 AGCAGAACAT TACTCACATC GAGAGCTCAA CCTATGCCTC CCTGCCTTCC AAATCACCAT

38641 CAAACTGGAC CTAAAAAGAT CATCCACCTA CTGGATTCTC TACAGCCACT GCCGTTGCAA

38701 AGACCCATAC TCGTTGTTTT GTCGAGCGCT TAATCAATAC GTGGCCCAGC AATGGCGATT

38761 AGATGTACGG GAGCACCTCG CCTCCGTACC CATACGCCAT CCCATCTCCA TTCGCGCTTA

38821 TGCTAAAAGG CGCACAGGGC ACTGCAGCCA CTGCACTTTC CACACGATCT ACGAAATTCT

38881 CACCGAGATC GTCAAACAAA CCTTCCAAGG GCGAAGTATC GTCATCTTTA GGAGACAAGA

38941 GGGTCGCATT AGATTAGGCA TCCCAAAATT TTTTCGCGAC TACGTCCACC TGTCCTGCTT

39001 CCTTAAAACC CTCGAAATTC TGCCACACAG CATTCATGTC TATTATTACT AATCTCTTGC

39061 AGACCACACG ACGGCCCACT TCCATCTTTC GCTGGACACC CGAAGACGAT CAAGTACGAC

39121 ACTCTGAACA CAGTTTCACC ATCAGAACCT ACGACCTCTT CGATGTCATA AACATACAAA

39181 TGCTATTCGA CCACCGATTC GTAGAGTTCA TGAACGGTGA CATTCAATTC CCCTTCCCGG

39241 GTCCCCACGA GTATTCACAA ACGAGCCTTT ACCCTAGAGT TAGACCGTTC AGAGCAGACA

39301 TCTCTGTTTA CTGTGTTCTC AGACCGTCCG AGGATACCGA ACTCGGATGG ATAGTAAAGC

39361 TGCGTTGCAA TTGCGGAGAC GGCAATTCCC TATTTTGTCA GTCTCTCAGA GAGCTCCTTT

39421 TCCACTCTTG GAAAGAAGCT CTACAAAACG GAGTCAGAGC AGAGCCCTTC CCCGTAGATC

39481 TAGGTCCCCT TACCTCGGAT GAACTAGGCC ACTGCGCATT TTGCACGGGA GCTACGCCTC

39541 TCGAACTAAT CTCAGAAATA GTACAGCATT GTCATAACGA TGGTGTAGCG CCACTCTCCA

39601 TCGAAGACGG TGAAATCATC CTCAGGCTTT CAGAATCCGC CAGCCAAGCC ATTAACCTTC

39661 CATGCTTCAT GCATTACATG CACAATTCTT TCCCCTACCC AACGCACATA CACACTAGGT

39721 AATCCTTCTC GTTGCAGATA CCCTCCGAAG AACTACACCC TGTATTCATT CACAAACGAT

39781 CTGACCGCAA CGGGCACGTC TCCCATATCA TTTCCATGCC TCCTATTCAC TCTGTGTCAT

39841 TCCAAGTCCT CACGAGTAAA GAATTTTTCG CCTTCTTTGT GGGAAAGTCT CAGTTTTTCA

39901 CTCCCGGAAC ACCCAATTCC TTTCAGGAAT TCGTTTTCTA TCCTCGATTC AAATCATTCC

39961 GAGTGGAAAT ACGTCTACGC GATATCAAAG CCGACGAAAC CAAAAACTGT GCAACCAGAT

40021 GGTTGCTCAA ATACCGGTGT ACCTGTCCCA AACCTCATAG TCTCTTCTGC CATTCCCTCC

40081 GAATGAAAAC CTATATACGT TGGGTTGACG AAATAAGAGC GACTACACGC GAAATCCCAC

40141 TCCCGGTACA TCTGGGCATT CTCGCCCATA GCTACCTAGA TCACTGCCAT GCGTGCAAGG

40201 ACGAAACACT TCTATTCATC TTGATCACTG CGGTTAATCC CAACAATGAA CCCTACCTCG

40261 AAATATCACA AACCCCATTC CGAGTTTCGC CAAAGGGGGT GCAAATATGC ATCCCGAGGA

40321 TCAAGAAGGA CGCTTTTCCA TATAGGTGCT TAATACGAGC CATGGAAATC TACTTTATTA

40381 ATTATACCCT CATTTTCTTT TAACAGAAAA TTGTTTTCCA CTCCAAGACT ATCCAACTTA

40441 ATCACCTTTT CGAACGAATC CCCTCCCATC TGGCCTTCGA CTCGCATTTC TTAAACTTCC

40501 TCGTGGGTCA ACGCATGTTC CCGTTCTGCC GTCGAGACTA CGTGAGTGCA GACGAACTAT

40561 TTCCCAAACT CGAGATGCCC CCTTTCAAAG TCAGACTGCG CCTCATGAAC TTTTATCCCT

40621 ACACGACTGA TCACGGTGCT CCCGCCATCC GATCCGAATG TTCCTGCGGA ATGCCCCATT

40681 CCCTGTTCTG CGAGAGCCTA GGCCAACTCG TATTCGCCTA CTGGTTCGAA ACCATTCAAG

40741 AATTTATCGA AGAACACATC CCCATCGATC CCTTCCCTCA CTGCCCCATC GAAGACATCA

40801 TCATGTGTCG TTGCAGTCTG CGCAAAGAGG GTCACTGCAA CCCGCTCTGG GGCTTACAAT

40861 TACTCTTCTG CAAAAGATCC ATTCTCGAAA ACTTAAGCAT CGGCCTAACC TCGACCGGCT

40921 ACAGAATCCT GATCCTCCCC AAATATTTCA GCCCACGAGT AAAAGCCGCA TGCGAACAAA

40981 TACAGAGAAA CTTGTCTCAA TTGAAGTTTA CTATTCAGAT TGAATGTTTT CCATTTTACA

41041 AATTTGAATA AATTACAACA TGTCTTCTGC ATTTATGTGT GGATTATTGC TAGCCCAGGT

41101 TACAAATTTA CGCGCGTCAT TACAAGTAAT AACAAAACCG GGTTCCACGC GTTCAGTCAA

41161 ACGAAGCACA TACATAGCAC TCCTCAGGGA ATTAGTATGA TCCTTACAAG TTTGGCCATA

41221 TTTCACATAG GTCAACGTTA CATCGTGACT CGTAGAAATG AGTACGGGGG TACGGTTAAT

41281 CTCCCGCCAT CTACGTTCTC CCGTCGCACC CTCCTCCACC TCCACCATAG TTCCCTCCCC

41341 TCTCAACACT TGCTCACACA ACCCTATCGC GCTCTCAGGG AAAAGCCCAC CATCTAACCA

41401 ATACACCAAA CAATTATAAT TGCGCGCAAA AGGATTGGCC CTATTCCGCC AATCCACACA

41461 TCCGACAACA GGAGAGGTAT AAGCTATTGC CTCTGCCAAA TAGGGCCCTC CCGTTTCTGC

41521 ACCTCCCCAC ACCCACACAG TATTCTTATT TGATGTAAGC ATGGACCACT TATGCAAAAT

41581 TGTTCCTACG ACACGGGGGT TGTAACCGTT TTTCATCAGA ATTACATAAA ACGGATTCAG

41641 ATGAATGTCG TCTTGGCACA CGACAGGGCA TACTAAGTAG TCACCGAGGA CTTTTGTCCA

41701 GCACATGTGT TTAATAACAT CGCGCAAAAC TTTGCGTACG CTGAACCCCC TAACGTATCC

41761 CTTCGAGTAC CGACGATATT CAGCCAAATC CGCAGATTGC CATTGTTCTC GCGTCACGAT

41821 GCCCCTGTTT ATTAACGAGT TCACTAAATC CCAGTAACGG TCCGAAGCCA TCTGTAATGC

41881 GAGCACATTT AATACATTTC AATCAGTTAC AATCAGTTAC AATCAGTTAC AATCGGTTCC

41941 CGAATCGCGG TCGCGGTCGC TGTCTTCAAA GACTTCTTCT TCCCCGCGCT GAACCGGAGC

42001 AGCGTTACGG TTCTCATCTA GCATCGCAGC CAGTTCATCA GCCGCGCGTG AAAGGGCATC

42061 GAAGTCCGGC ATGGACATAA ATCGCAGTCG AGGCCGACCG CGGAAGTCAT CCCAGGCGTT

42121 AAGCAGCTCG GCAACAAGCA TCCAAGTGCC GACAAGCATC GCGGTGGGCC AGTGAACACC

42181 GAGAGTATGA AAGATGTAAC AATCTACAAC AATCAAACAG AAGAGAACTA GAATCTTAAG

42241 AAACATACCT AAAGAATTAC ACACGGAGAA CAACTACAGA GAGAGCAAGA ATTTCTTCCA

42301 GTATCCCCCT CGCAAAAACA ATTCGTGTAA GCGTCGTTAT CTGCCTCCAC TTCTGCGGAA

42361 ATCTTCACCG CAAAGTCGGG TTCGTCGAAA GCGCGTTCGT GACACGCAGA ATCAGCCTAC

42421 GAAGAAGAAC CAGTACCGTT GGGTTTAAGC CACAAATGTA GAGTCCTTTG AGAGGACATC

42481 GCTTCACTAC CAGTGCCAGT CGACCCGAAT CCGTTCCCTC CTCGGATCGT GTCATCAACG

42541 GAGGACACCT CCTCGAGCTC CGGTGTAAAG ATTCGCTCAA CAACGAGCTG AGCGATACGG

42601 TCTCCCTTCC GAACGTTGAA CGCATGTTGC GAAAAATTGA ACAAGAGCAC CTTCACTTCA

42661 CCGCGATAAT CAGCATCTAT CACACCCGCC CCCACATCTA TAAAGAACTT TGCAGCCAAT

42721 CCCGAGCGAG GAGCGATTCT CCCATAAGTG CTGGGAGGCA GGATTAAAAT TAACCCCGTA

42781 GAAATCAGCG CCTTGTCGTG AGGTGGTACC ACTACGTCCT CACAACTAAA CAAATCTAAA

42841 CCCGCTGCAT CTGGCGACAT GCGGACAGGA GCGAAAGCAT CCGATTTAAC CTTCTTATAT

42901 AGAAGTTTAG GCACAGACGG AGACGCAGAA ACGGCAGCCA TCCTTGTAGA AAAAGAGAGA

42961 AAATTGTCAA GGGATGACAC CACCGCGAAA AACGTACAGA AAACAATCCT CACCTATAAC

43021 AGTTCAACGA TCTTCTCCAA CGCCGCGTTC AGTTCACTCT GCTTCACGGT GCCGTTCCAG

43081 AGGATTTCGG TCCAAAACCG GTTGGGTTCA AAGCAGTGGA GGAATACACG CCGAGGCGTA

43141 ATAGGGTGAA ATTGAATTTC AAAGTTATGG CAAGGCTCGC TGATCGCGAT ATCGGCGTTT

43201 TCAGCGTCAA GCAATGCACA GCGAGTAAGA GGGACAGTTC TCCTATAGCT CATAGGAAGA

43261 TCTGTAACGA AAGGACGGAC TCGCCAGTAC TTATATCGTG AGGACCGTCC CTTAAACGGA

43321 GTGGAAAAAT ACCTTTCGTG ACAACGGGTA ATGGTATCTG ATCGATATCT AAACTCGTGG

43381 TGCTATTAAG GCTGTCCCTT GACCCGCAAT TAGTACAATT ACTGACCGAA GTACACTGAA

43441 ACGTAAATAT TTAACACAAA CAAGCGAATA AAACATGACC TTCAAACTTG CAACGAACGG

43501 TAAACATTTC AAGGATATGG GATGTACCGG GAAAAACACT TAAATTACAC ATCACGTTAG

43561 GACTTTGCCC AGGCGTTGCC AGTTTCGAAC TACACGCGAA AGCGAAACTG GCGTCATGAG

43621 ATCAAAGTTC AGAACCCTAG TGTGCAAACC CGGAAATTCA TGCAATGACT CAGCCGCGCT

43681 GCGCGCGGCG GTTGTAAGTG TGTCAAAAGA CGCGGTTATA TAAGATGATG AGATCGGA
